# Dynamics of archaea at fine spatial scales in Shark Bay mat microbiomes

**DOI:** 10.1038/srep46160

**Published:** 2017-04-11

**Authors:** Hon Lun Wong, Pieter T. Visscher, Richard Allen White III, Daniela-Lee Smith, Molly M. Patterson, Brendan P. Burns

**Affiliations:** 1 School of Biotechnology and Biomolecular Sciences, The University of New South Wales, Sydney, Australia; 2 Department of Marine Sciences, University of Connecticut, USA; 3 Australian Centre for Astrobiology, University of New South Wales Sydney, Australia; 4 Earth and Biological Sciences Directorate, Pacific Northwest National Laboratory, Richland, Washington 99352, USA

## Abstract

The role of archaea in microbial mats is poorly understood. Delineating the spatial distribution of archaea with mat depth will enable resolution of putative niches in these systems. In the present study, high throughput amplicon sequencing was undertaken in conjunction with analysis of key biogeochemical properties of two mats (smooth and pustular) from Shark Bay, Australia. One-way analysis of similarity tests indicated the archaeal community structures of smooth and pustular mats were significantly different (global R = 1, p = 0.1%). Smooth mats possessed higher archaeal diversity, dominated by Parvarchaeota. The methanogenic community in smooth mats was dominated by hydrogenotrophic Methanomicrobiales, as well as methylotrophic Methanosarcinales, Methanococcales, Methanobacteriales and Methanomassiliicoccaceae. Pustular mats were enriched with Halobacteria and Parvarchaeota. Key metabolisms (bacterial and archaeal) were measured, and the rates of oxygen production/consumption and sulfate reduction were up to four times higher in smooth than in pustular mats. Methane production peaked in the oxic layers and was up to seven-fold higher in smooth than pustular mats. The finding of an abundance of anaerobic methanogens enriched at the surface where oxygen levels were highest, coupled with peak methane production in the oxic zone, suggests putative surface anoxic niches in these microbial mats.

Microbial mats in extreme environments provide insights into how physiochemical stresses shape adaptive response in both individual organisms and entire communities. Microbial mats are found globally in environments such as acidic mines drainages^1^, remnant asbestos mines^2^, cold freshwater lakes^3^,^4^ and hypersaline environments in Guerrero Negro^5^,^6^, Kiritimati Atoll^7^, the Bahamas^8^,^9^,^10^,^11^, and Shark Bay, Australia^12^,^13^,^14^,^15^. Microbial mats in Shark Bay are one of the best examples of modern living microbial mats, and have been proposed to be analogous to Precambrian stromatolites^16^,^17^,^18^,^19^. The extreme conditions of hypersalinity, desiccation stress, and high UV irradiance observed at Shark Bay, may be similar to conditions present on the early Earth^20^,^21^,^22^,^23^. Therefore, it is proposed that living microbial mats can provide us with insights into the evolution of the early biosphere^24^,^25^.

Microbial mats consist of diverse microbial ecosystems that are formed by multidimensional interactions between biotic and abiotic factors that select for unique functional groups^26^. As a result of these interactions, critical nutrient and biogeochemical cycling (carbon, oxygen, nitrogen and sulfur) usually occur at a millimetre scale and fluctuate during a diel cycle^5^,^6^,^27^,^28^, thus imposing steep chemical gradients within fine spatial resolution^29^,^30^. This favours niche differentiation among microbial communities, and can also reflect the spatial nutrient requirements and cycling within the mat systems^6^, and potentially how microorganisms interact with each other and the surrounding environment^31^.

Resolving the taxonomic and functional diversity, complexity, and assembly of microbial mat systems has been made possible by employing a combination of classical geomicrobiological techniques with modern next generation sequencing approaches^13^,^28^,^32^. However, despite an increasing number of studies indicating archaeal prevalence in some microbial mat systems^6^,^13^,^25^,^33^, the overall role(s) of archaea in microbial mats remains poorly understood. Interestingly, archaea appear to be more abundant in the Shark Bay microbial mats compared to those in open marine ecosystems in the Bahamas^13^. Although early work in Shark Bay indicated microbial mats were dominated by Halobacteria^12^,^14^,^34^,^35^, the techniques used in these studies likely under-represent the true archaeal diversity in Shark Bay mats. Furthermore, new archaeal classifications have been proposed^36^,^37^, and revision of the classification with closer link to physiologies would help linking functional potential to taxonomic markers such as the 16S rRNA gene, thereby refining and establishing ecological roles of archaea in these environments. Finally, a caveat of many studies relying solely on 16S rDNA data is that there are limits to how much function can be inferred from phylogeny, and thus conducting defined metabolic measurements to support molecular analyses is critical to facilitate a more comprehensive understanding of mat ecosystems.

A recent study deciphered for the first time bacterial community distribution, abundance, and putative interactions at the millimetre scale in Shark Bay microbial mats^28^. Employing iTag deep amplicon sequencing coupled with biogeochemical measurements, the spatial distribution of bacterial groups with mat depth and patterns of niche differentiation were inferred. However, complementary analyses resolving the archaea at a discrete millimetre scale are needed to fully characterise the microbial communities in Shark Bay. Therefore, the aim of this study was to characterise the spatial distribution of archaea with depth combined with defined metabolic measurements, in order to elucidate putative ecological niches and microbial functional roles of this domain in Shark Bay microbial mats. In the current study, deep iTag amplicon sequencing of archaeal small subunit RNA, coupled with biogeochemical measurements (O_2_ production and consumption, sulfate reduction, and methane metabolism), were undertaken in microbial mats in Shark Bay. This study has greatly enhanced our understanding of archaeal community dynamics in microbial mat systems, and their putative roles in potentially ‘filling the niches’.

## Results

### Biogeochemical measurements in Shark Bay microbial mats

**Figure 1:**
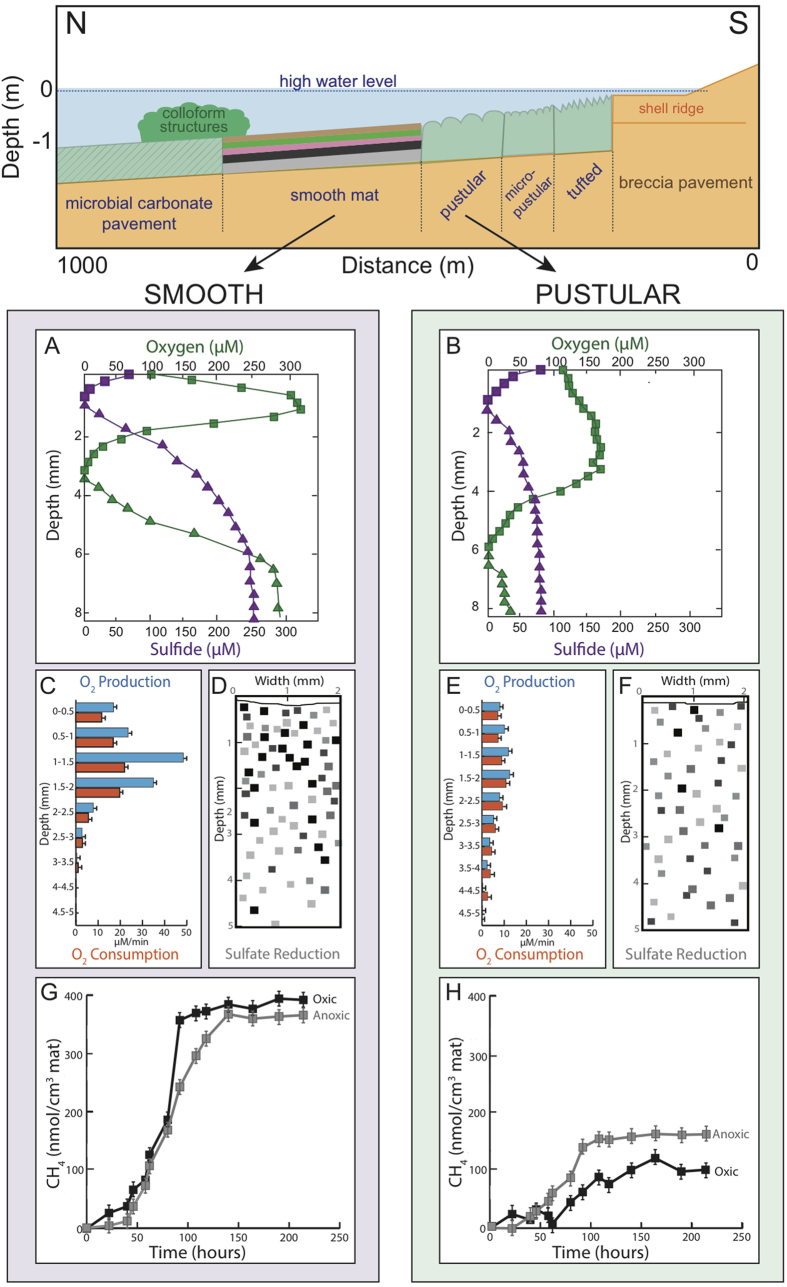
Nilemah transect showing position of microbial mats and metabolic rates measured. *Top panel:* schematic showing transect with tide and mat position of the microbial mats investigated in the present study. (**A**) and (**B**) *In situ* depth profiles of oxygen and sulfide concentrations in Shark Bay microbial mats ((**A**), smooth mats; (**B**), pustular mats) measured with needle electrodes. Oxygen and sulfide concentrations were measured during peak photosynthesis between noon and 2:00 pm (green symbols and lines) and the end of the night, between 4:00 and 5:00 am (purple symbols and lines). Squares represent oxygen concentrations, triangles represent sulfide concentrations. (**C**) and (**E**) Depth profiles of oxygen production and consumption in Shark Bay microbial mats ((**C**), smooth mats; (**E**), pustular mats). Oxygen production and consumption were measured *ex situ* using the light-dark shift technique. Blue bars = oxygen production, orange bars = oxygen consumption. (**D**) and (**F**): Two-dimensional distribution of sulfate reduction in Shark Bay mats visualized using the silver foil technique ((**D**), smooth mats; (**F**) pustular mats). Trace near the top of the panels indicates the surface of the mats. Pixels indicate locations of sulfate reduction activity; darker pixels represent higher rates. (**G**) and (**H**): Time series of methane production in unamended Shark Bay microbial mat slurries ((**G**), smooth mats; (**H**) pustular mats), expressed as methane produced per cm^3^ mat used for the slurry preparation. Symbols represent average of three replicate bottles. Error bars represent ± one standard deviation.

Smooth mats displayed a shallow O_2_ maximum of 321 ± 26 μM at 1.25 mm depth (average ± standard deviation; n = 3) and a relatively shallow oxycline of 3.2 ± 0.1 mm depth even during peak photosynthesis (light intensity 1780–2040 μE.m^−2^.s^−1^; Fig. 1A). Oxygen penetrated deeper in pustular mats (i.e., down to 6 mm) than in smooth mats, but the maximum concentration of O_2_ was two-fold lower (175 ± 14 μM), with a broad O_2_ maximum between 1.5 and 3.75 mm depth (Fig. 1B). The oxygen production and consumption rates were higher in smooth mats compared to pustular and concentrated near the surface (upper 3.5 mm). Similarly, sulfate reduction rates, as evidenced by pixel densities indicating Ag^35^ S distribution (Fig. 1D,F), were higher in smooth mats than in pustular mats. Furthermore, the bulk of the microbial activity was concentrated in the upper ca. 3 mm in smooth mats compared to the upper 5–6 mm in pustular mats. Compared to smooth mats, the sulfide concentration at 8 mm depth was 3–7 times lower in pustular mats. The independent metabolic profiles obtained on the mats in the present study corroborated very well with recent measurements taken during the analysis of bacterial populations in these ecosystems^28^.

### Estimation of richness and diversity

A total of 13,547,552 unfiltered sequences were obtained. To account for sampling bias, databases were subsequently subsampled so that each sample contained 31,000 sequences, with a total of 1,147,000 sequences in all the samples. Rarefaction analysis and non-parametric estimators were performed to determine archaeal diversity and richness. Alpha diversity analyses of all samples were performed at 97% evolutionary distance level of OTUs. Rarefaction analysis showed that the curves have not yet reached an asymptote (Supplementary Figure 1), indicating further sampling is needed to reach saturation of richness. Shannon indices ranged from 4.51 to 5.67 in pustular mats and 3.98 to 5.67 in smooth mats (Supplementary Table 1). Both mats show the highest archaeal diversity in the bottom layer.

### Overall archaeal community composition

**Figure 2:**
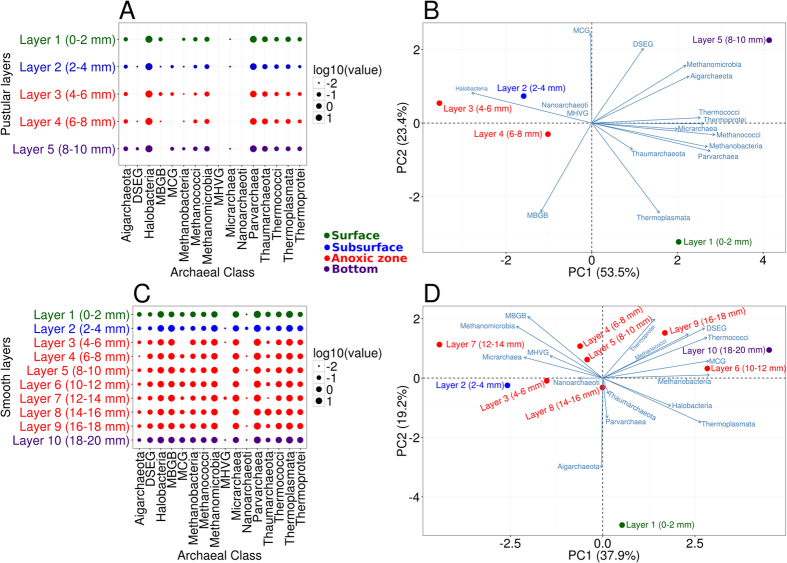
Archaeal community composition in Shark Bay microbial mats with depth. (**A**) Archaeal distribution (class level) in pustular mats at 2 mm depth intervals. (**B**) PCA biplot of Shark Bay pustular mats microbial community profiles from different depths. Blue lines indicate the direction of increased taxon abundance at class or order level, and the length indicates the degree of correlation of the taxa with community data (**C**) Archaeal distribution (class level) in smooth mats at 2 mm depth intervals. (**D**) PCA biplot of Shark Bay smooth mat microbial community profiles from different depths. Blue lines indicate the direction of increased taxon abundance at class or order level, and the length indicates the degree of correlation of the taxa with community data. MBGB - Marine Benthic Group B; DSEG - Deep Sea Euryarchaeotal Group; MCG - Miscellaneous Crenarchaeotal Group; MHVG - Marine Hydrothermal Vent Group.

Smooth mat archaeal communities were dominated by *Parvarchaea* (38.67%), *Thermoplasmata* (19.99%), MBGB (9.74%), *Methanomicrobia* (10.17%), *Micrarchaea* (7.84%) and *Halobacteria* (6.91%). In contrast, pustular mats were dominated by *Halobacteria* (72.67%), *Parvarchaea* (25.03%), and *Thaumarchaeota* (1.3%) (Fig. 2C, Supplementary Table 2). One-way analysis of similarity test (ANOSIM) indicated the archaeal community structures of smooth and pustular mats were significantly different (global R = 1, *p* = 0.1%). In particular, archaeal classes MBGB, Thermoplasmata, and Methanomicrobia were abundant in smooth mats but virtually absent (<0.5%) in pustular mats. The recently proposed Parvarchaeota dominated smooth mats and was also abundant in pustular mats.

### Vertical distribution of archaeal communities in smooth mats

Smooth mat surfaces were highly enriched with *Parvarchaeota* order YLA114 (42.39% at the surface), followed by *Thermoplasmata* order E2 (26.37% at the surface) (Supplementary Figure 2). E2 was characterised by archaeal family CCA47, DHVEG-1 (Deep sea hydrothermal vent group I) and Methanomassiliicoccaceae. YLA114 and CCA47 gradually deplete with depth. In contrast, *Parvarchaeota* order WCHD3-30 and *Thermoplasmata* lineage DHVEG-1 increase with depth and peak at the bottom layer (15.92% and 24.41% respectively) (Supplementary Figure 2). *Crenarchaeota/Thaumarchaetoa* class MBGB peaks at the subsurface (19.42%) (2–4 mm), then gradually decreases with depth. *Euryarchaeota* was primarily represented by members of class *Thermoplasmata*, which was also the dominant member at the bottom layer. Hydrogenotrophic methanogens Methanomicrobiales comprise over half of the total methanogenic community, which peaks at the subsurface (2–4 mm) and gradually decreases with depth. Conversely, other known methanogens such as *Methanosarcinales, Methanococcales, Methanobacteriales* and the recently classified *Methanomassiliicoccaceae* are methylotrophic, and gradually increase in abundance with depth in smooth mats.

To examine phylogenetic stratification by spatial layers in the mats, cluster and PCA analysis were undertaken. Smooth mats were phylogenetically stratified into distinct spatial mat groups by layer in the mats, primarily surface (0–2 mm) and bottom layers (18–20 mm), and some clustering of subsurface (2–4 mm) and anoxic zone (6–18 mm) (Fig. 2D). STAMP analyses were employed to identify which archaeal members were overrepresented in the mat layers^38^. The surface of smooth mats was enriched in *Parvarchaeota* order YLA114 (42.39%) and *Thermoplasmata* family CCA47 (11.47%) (Supplementary Figure 3), while the subsurface was characterised by *Crenarchaeota/Thaumarchaeota* class MBGB (19.42%) and the hydrogenotrophic *Methanomicrobiales* (10.61%) (Supplementary Figure 4). The bottom layer of smooth mats was represented by various methylotrophic methanogens, *Parvarchaeota* order WCHD3-30 (15.92%) and *Thermoplasmata* lineage DHVEG-1 (24.41%) (Supplementary Figure 5). The anoxic zone (6–18 mm) had an even distribution of archaeal diversity with no group significantly enriched.

### Vertical distribution of archaeal communities in pustular mats

In contrast, to smooth mats, pustular mats had >60% of the archaea represented by *Halobacteria*, followed by *Parvarchaeota* (Fig. 2A). When compared to smooth mats the pustular mats had very low levels of methanogens by sequence analysis (<0.5%). Halophilic archaeal genera dominanted pustular mats namely, *Halogranum* (10.48%)*, Natronomonas* (17.41%)*, Halorhabdus* (15.98%)*, Halobacteriaceae* (5.98%) and *Halorientalis* (0.72%). A candidate genus ArcF12, which belongs to the family *Halobacteriaceae*, was also found abundant in pustular mats (16.61%) (Supplementary Figure 6). The genus *Nitrosopumilus* was the dominant Thaumarchaeota in pustular mats, which is an active nitrifier. Pustular mats revealed only two groups in regards to phylogenetic clustering, namely the top layer (0–2 mm) and the subsequent layers (2–10 mm) (Fig. 2B). STAMP analysis revealed that none of the archaea in pustular mats was significantly enriched in any layer.

### Methane production

**Figure 3:**
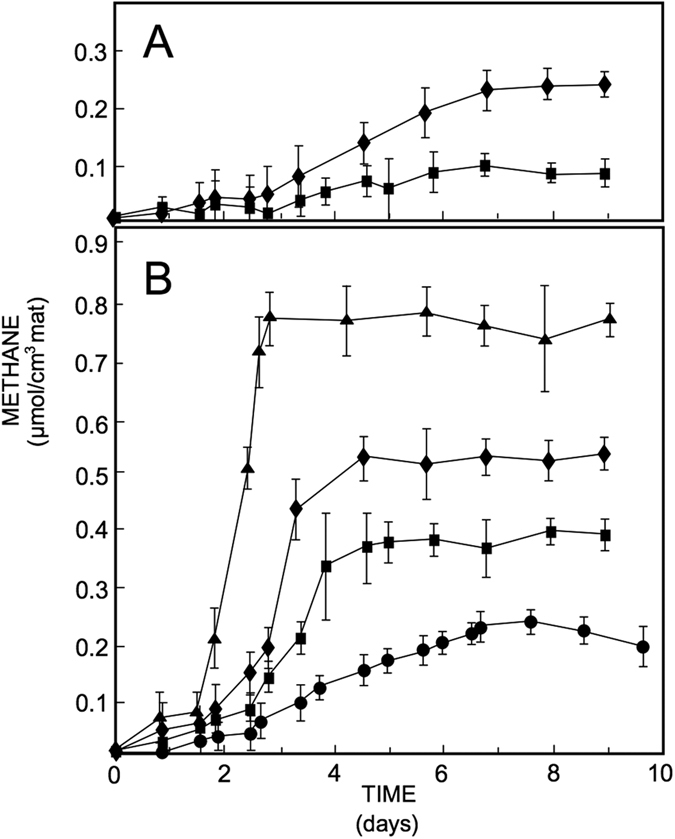
Methane production in slurries prepared from the surface layer (oxic part) of Shark Bay microbial mats. (**A**) Pustular mat. (**B**) Smooth mat. Symbols used in both panels: squares = control (no additions); diamonds = molybdate; circles = headspace replaced with air daily; triangles = H_2_/CO_2_ headspace; methane production is expressed per cm^3^ mat used for the preparation of the slurries. Symbols represent average of three replicate bottles. Error bars represent ± one standard deviation.

Time series of CH_4_ production (nmol CH_4_.cm^−3^ mat) in smooth mat slurries showed no or only a short (i.e., less than 12 h) lag phase before the headspace concentration of CH_4_ started to increase (Fig. 1G,H). Slurries prepared from the oxic part of smooth mats showed the shortest lag phase and accumulated the largest amounts of methane. In contrast, slurries prepared from pustular mats did not produce methane until 12–24 h, suggestive of a smaller active methanogenic population present in pustular compared to smooth mats. When methane concentrations levelled off, potential methane production was calculated from the increase in CH_4_ with time. In unamended slurries, the rate of methane production (nmol CH_4_.cm^−3^ mat.h^−1^) was six to seven times higher in smooth than pustular mats, which correlates with the putative lower abundance of methanogens detected at the molecular level in pustular mats described earlier. The rates were comparable in the oxic and anoxic parts of the smooth mat and only slightly higher in the slurries of the anoxic part of pustular mats compared to those in the oxic part (Fig. 1). In slurries of smooth mats, addition of methanogenic substrates resulted in an increase in methane production rate (nmol CH_4_.cm^−3^ mat.h^−1^), with the greatest effect of H_2_/CO_2_ (Fig. 3). Addition of excess H_2_/CO_2_ to smooth mat slurries doubled the rates of CH_4_ production and was more effective than addition of excess trimethylamine – a non-competitive substrate.

Inhibition of sulfate reducing bacteria with molybdate increased methane production rates by almost half and inhibition of methanogens using bromoethane sulfonate (BES) or autoclaving reduced activity completely (data not shown). The methane production potential in slurries prepared from the oxic part of smooth mats appeared slightly higher than those in slurries prepared from the anoxic part of these mats (Fig. 1). In pustular mat slurries, the patterns of potential methane production were similar: addition of exogenous substrate increased the rate of CH_4_ production up to three times, and inhibition of sulfate reducing bacteria doubled the CH_4_ production rate. The rate measured in slurries prepared from the anoxic part of the pustular mats was always higher than in slurries prepared from the oxic part of these mats. When the headspace over the slurries was replaced by air daily, the CH_4_ production rate decreased to ca. half in slurries from both oxic and anoxic parts of the smooth mat. In pustular mats, this pattern was the opposite with anoxic slurries having a higher potential methane production rate than oxic slurries.

## Discussion

This study describes for the first time the spatial distribution of archaea at high resolution with depth in distinct Shark Bay microbial mats, through a combination of high-throughput 16S SSU sequencing of community DNA and *in situ* measurements of major metabolic pathways, including oxygenic production and consumption, sulfate reduction, and methanogenesis.

### Physiochemical conditions affecting microbial community structures

Smooth and pustular mats in Shark Bay are found in the littoral zone and are exposed to the atmosphere each tidal cycle. During this period, mats increase in temperature, salinity of the water film overlying the mats increases rapidly, and in certain cases (e.g., high wind), desiccation may set in. Therefore physicochemical conditions inside mats are likely subject to extreme changes at least twice daily. As such, this represents a stressed environment whereby pustular mats are more exposed than smooth mats due the relative location along the tidal transect (Fig. 1). The difference in physicochemical conditions in mat microenvironments - in particular salinity possibly shaping community dynamics - may contribute to the different archaeal diversities observed, as well as the distinct metabolic profiles present in a given mat system (i.e., higher sulfate reduction rates, and higher organic carbon and methane production in smooth mats). The lower overall diversity (albeit higher Halobacterial diversity) observed in pustular mats compared to smooth mats, is thus suggested to be the result of differences in location between the two mat types in the tidal transect of Shark Bay and the use of potentially unique osmoregulation strategies^39^,^40^.

### Archaeal groups ‘filling the niche’ in Shark Bay microbial mats?

Previous work on the Shark Bay mats suggested that the Thaumarchaeota may be ‘filling the niche’ in terms of nitrification^28^, as common bacterial nitrifiers and their pathways are in low abundance in the Shark Bay systems^13^,^28^. This may be particularly relevant to nitrogen cycling in Shark Bay, as high salinity has been shown to inhibit bacterial nitrifiers in other systems^41^. The detection of the known archaeal nitrifier *Nitrosopumilus* in the present study may help support this hypothesis, however further investigation comparing archaeal and bacterial nitrification in the Shark Bay systems is needed to definitely delineate the roles of these two groups in nitrogen cycling. Without ammonia oxidisers, accumulation of ammonia is detrimental to microbial ecosystems and therefore Thaumarchaeota such as *Nitrosopumilus* are proposed to play a role in oxidising ammonia. Potentially this could be facilitated through degradation of glycine betaine^42^,^43^ – an osmoprotectant common in Shark Bay - or deamination of proteins from decaying microbial mat biomass^44^. Whether any microorganisms that possess comammox – a recently discovered pathway that oxidises ammonia to nitrate directly^45^ – are present in Shark Bay mats remains to be determined.

### Hydrogenotrophic methanogens

Interestingly, under the conditions of the present study, a wide diversity of methanogen sequences was detected in smooth mats, including orders *Methanomicrobiales, Methanosarcinales, Methanobacteriales and Methanococcales. Methanomicrobiales* is represented here by the genus *Methanofollis*, which is strictly hydrogenotrophic and anaerobic^46^. *Methanomicrobiales* comprises over half of the methanogenic population in smooth mats, suggesting that hydrogenotrophic methanogenesis may dominate methane production in smooth mats. Addition of excess H_2_/CO_2_ or trimethylamine to mat microcosms in the present study increased rates of CH_4_ production, corroborating the importance of hydrogenotrophic and methylotrophic methane production. The observation of relatively low abundance of methanogenic sequences in pustular mats was supported by the observation that these mats only started producing methane after a lag phase, and that methane production was significantly lower than in smooth mats (Fig. 1). However, inhibition of sulfate reducers stimulated their activity, and thus methanogenesis still likely has a role in pustular mat systems.

The apparent dominance of hydrogenotrophic methanogens in Shark Bay smooth mats is in contrast to findings in other mat systems, where methylotrophic methanogens generally predominate^47^,^48^,^49^,^50^. However, our study agrees with another study that demonstrated methanogenic communities are vertically compartmentalised in hypersaline microbial mats^33^. Methanogenesis is typically dominated by methylotrophic methanogens rather than hydrogenotrophs in hypersaline settings, as sulfate-reducing activities usually outcompete the latter for hydrogen thermodynamically^33^,^49^,^51^,^52^,^53^,^54^,^55^,^56^, especially at salinities below 195 ppt^57^. Indeed high sulfate reducing activity was recorded at the surface of Shark Bay smooth mats here and in other studies^28^,^58^, which one would expect to restrict the hydrogenotrophic Methanomicrobiales. It is hypothesised that the subsurface of Shark Bay smooth mats holds a putative niche – or trophic strategy - for hydrogenotrophic methanogenesis, where *Methanomicrobiales* peaks potentially due to its proximity to a putative hydrogen source.

### Methanogenesis under hypersalinity

Previous studies in Shark Bay mats have described the importance of the accumulation of osmoprotective molecules, in particular glycine betaine, as adaptive mechanisms to counter the high salinity conditions^13^,^39^,^40^. Osmoregulation is likely a critical aspect of Shark Bay mat community ecophysiology. The addition of trimethylamine, a non-competitive substrate for methanogenesis^52^,^55^ and a degradation product of glycine betaine^42^, stimulated methane production 1.6–1.8 fold in smooth mats and 2.4–3.0 fold in pustular mats in the present study. Furthermore, some methanogens in other ecosystems have been shown to even utilise glycine betaine as another alternate non-competitive substrate for methanogenesis^59^. Thus it is hypothesised here that as a result of salinity stress Shark Bay mat communities may need to produce high levels of glycine betaine and other methylated compatible solutes as osmoprotectants, which can then also be potentially utilised for methanogenesis. Although further work is needed to clarify this, such a model would facilitate a competitive advantage for archaea to undergo methylotrophic methanogenesis in these systems.

### Co-existence of sulfate-reducing bacteria and methanogens

Despite sulfate-reducing bacteria (SRB) usually outcompeting methanogens thermodynamically, both co-exist at the subsurface in microbial mats^27^,^28^,^57^,^60^. SRB is represented by the order Desulfobacterales in Shark Bay mats^28^, which was found to be a primary hydrogenotroph in a hypersaline microbial mat in Elkhorn Slough, USA^61^. This group of SRB also peaked at the subsurface (2–4 mm) in smooth mats^28^ – and correlated here with measurements of sulfate reducing activity (Fig. 1) - and an early study in Shark Bay also identified that SRB in smooth mats were quantitatively important H_2_-consumers^62^. Though it appears that *Desulfobacterales* and *Methanomicrobiales* are competing for H_2_, the SRB and the hydrogenotrophic methanogens might be in a syntrophic relationship. High levels of H_2_ are proposed to cause end product inhibition of the carbon sources that methanogens need for growth^33^. Thus, it is proposed here that putative H_2_-consuming Desulfobacterales may act as a regulator to prevent excess H_2_ accumulation, enabling the dominance of hydrogenotrophic Methanomicrobiales in smooth mats.

The fluctuation of salinity facilitated by the tidal regime in Shark Bay may also play a role in the co-existence of SRB and methanogens. Salinity measurements taken at different tidal points in Shark Bay illustrate the increase of salinity during low tide (salinity increased beyond 195 ppt) as the mats were subjected to desiccation stress (Supplementary Figure 7). In hypersaline mats of Puerto Rico, methanogens outcompeted sulfate reducers at salinities above 195 ppt^57^. A similar scenario may be prevalent in Shark Bay during low tide, when the high salinity may impede the metabolic activity of SRB, potentially rendering substrate for methanogens. It is proposed here that high salinity may impede the metabolism of SRB, allowing methanogens to take over at the surface, while SRB metabolisms predominate when salinity drops below 195 ppt.

### Surface anoxic niche

The high rates of sulfate reduction observed at smooth mat surfaces (Fig. 1) indicate putative microzones of anoxia in oxic parts of the mat, which can further be exploited as possible niches for methanogens (shown by high methane production rates at the surface of smooth mats). Conversely, the lower rates of sulfate reduction in pustular mats (Fig. 1), suggested fewer putative anoxic microzones, which was also reflected in the lower methanogen diversity at the taxonomic level, and lower rates of methanogenesis observed. These putative anoxic surface niches were further supported by previous work indicating a high proportion of bacterial anaerobes at the surface of these mats in Shark Bay^28^. Another finding in the present study supporting a potential surface anoxic niche was the detection of abundant *Thermoplasmata* sequences (order CCA47) at the surface in smooth mats in Shark Bay, and previous studies have primarily detected these groups in oxygen-depleted sediments^63^,^64^.

These findings corroborate further the suggestion that there are anoxic/suboxic microniches at the surface of smooth mats^28^,^65^. It has been proposed that tightly regulated metabolisms – potentially mediated by quorum sensing – of microbial consortia such as cyanobacteria, sulfate reducers/sulfur oxidisers, and methanogens/methanotrophs, may create these oxic and anoxic zones at mat surfaces^66^. Further support for these putative cooperative niches was the finding that phototrophic sulfide-oxidising bacteria (e.g. Chromatiaceae) were significantly enriched at the surface of these Shark Bay mats^28^, in addition to putative methanotrophic bacteria (e.g Verrucomicrobia) in high abundance at mat surfaces^28^. Therefore, we suggest that niche differentiation and metabolic specialisation in the mats may be shaped by microbial interactions between functionally distinct groups (i.e. *Methanomicrobiales,* Cyanobacteria*, Desulfobacterales,* Chromatiaceae, Verrucomicrobia) and surrounding physiochemical conditions, leading to the spatially driven unique niches of microbial community structure by layer in Shark Bay microbial mats. The presence of these groups at mat surfaces may be key in efficient cycling of organic C, S, CH_4_, and O in these systems, and the maintenance of these putative anoxic/suboxic surface niches.

### Evolutionary consideration

The presence of hydrogenotrophic methanogens described in the present study and high levels of molybdenum (Mo) detected in these modern smooth mats^28^, indicates the systems in Shark Bay might be similar to their ancient counterparts in the Precambrian period^67^. It has been proposed that hydrogenotrophic methanogenesis is an ancient form of methane production^68^,^69^, whilst enriched Mo was also proposed as an indicator of ancient microbial mats^70^. Furthermore, it has been proposed that the origin of Mo-dependent nitrogenase is linked to a hydrogenotrophic methanogen^71^, suggesting smooth mats may have retained evidence of an ancient origin.

### Conclusions

**Figure 4:**
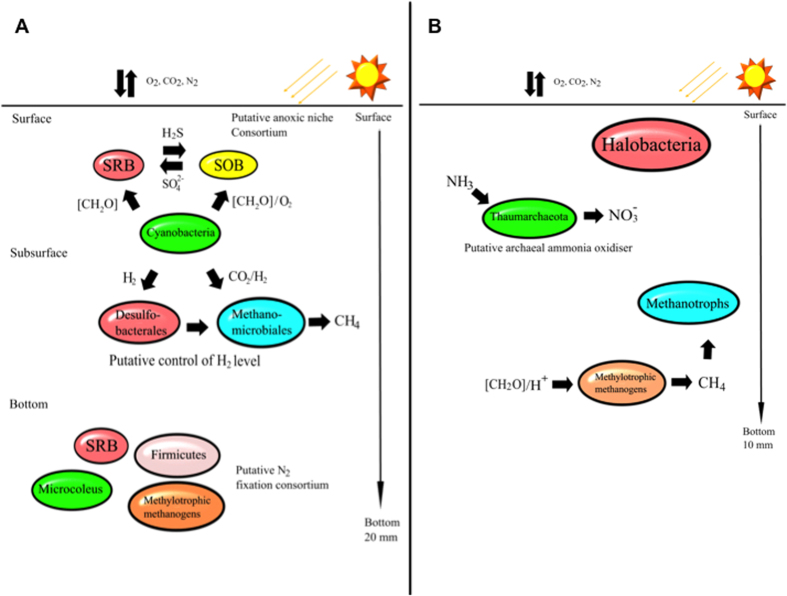
Schematic illustrating some of the key putative microbial interactions occurring in Shark Bay microbial mats inferred from the present study. Putative interactions are based on archaeal distribution determined by 16S rDNA sequencing and methanogenesis measurements, as well as previous work on bacterial distribution with depth in these microbial mat systems (Wong *et al*., 2015). (**A**) Putative key interactions in smooth mats (**B**) putative key interactions in pustular mats.

Findings in the present study suggest that archaea may be critical in ‘filling the niches’ and could be key players in Shark Bay microbial mats. A schematic summarising some of the key interactions inferred from this study is shown in Fig. 4. The present study delineates Parvarchaeota as the dominant archaeal group, though their exact role in ecosystem function is unclear. The smooth and pustular mats have significantly different microbial community structures, and hydrogenotrophic methanogens are enriched despite being potentially less favoured thermodynamically in the presence of sulfate reducing bacteria. Interestingly, it has been suggested that hydrogenotrophic methanogenesis is an ancient form of methane production^68^,^69^, and thus the modern Shark Bay mats may be good proxies for their ancient counterparts^67^. This high-resolution phylogenetic characterisation, coupled to data describing the spatial distribution of bacteria in these mats^28^, reinforces a putative model of a surface anoxic niche in these systems. It should be acknowledged that there are limitations to the present study, given the focus at the molecular level was on one taxonomic marker (16S rDNA of archaea) from total community DNA. To this end, future functional metagenomic and metatranscriptomic profiling at different depths (also targeting cDNA and active communities), coupled with microbial activity measurements (CH_4_, O_2_), along with techniques such as Nano FISH-SIMS, stable isotope analyses, and metaproteomics, are required to confirm the data and the putative niches and interactions proposed. In addition, although the differences of metabolic measurements between surface oxic and deeper layers is highly reproducible some of the differences between layers is small, and thus follow up studies over different depths (and seasons) are needed to ascertain true functional roles in these proposed niches. Nevertheless, our data suggests there is likely a reservoir of untapped archaeal diversity in these systems, and metabolic cooperation between key microbial groups is proposed to be important in efficient cycling of key nutrients.

## Materials and Methods

### Sample collection

Smooth and pustular mats were sampled on 16^th^ June 2013 from Nilemah, Hamelin Pool, Shark Bay (26°27′336″S, 114°05.762″E), using methods as previously described^12^,^28^. Of the two mats, pustular mats were located closer to the shore than smooth mats (Fig. 1). At the time of sampling, water temperature was 24.8 °C, salinity 68 PSU, and pH 8.13. Triplicate mat samples were taken using a sterile scalpel and placed in sterile containers. Half of the mat samples were immediately preserved in RNALater (Ambion, Life Technologies) for downstream DNA analyses. In order to determine the archaeal composition in successive mat layers, the samples were dissected into 2 mm intervals using sterilised blades as previously described^28^.

### Measurements of key biogeochemical properties

In order to compare major microbial processes between the two mat types, and to determine the approximate depth of the oxic and anoxic zones, depth profiles of oxygen, sulfide and pH were measured in small mat samples (~5 × 5 cm) submerged in 3 cm of water collected from the site. Profiles were measured in triplicate using needle microelectrodes^27^,^58^ *ex situ* under ambient temperature and light intensity within 1 h of sampling. Previous studies in this environment have shown that *in situ* and *ex situ* measurements under natural light were nearly identical^58^, and thus accurate reflections of conditions *in situ*. Needle electrodes with a tip diameter between 100 and 150 μm (Unisense, Denmark) and an outer diameter of the needle ranging from 0.55 to 1.0 mm were deployed in 250 μm depth increments using a manual micromanipulator (National Aperture, New Hampshire). Electrode readings were carried out during the peak of photosynthesis between 1130 and 1400 h. Polarographic oxygen and sulfide electrodes (Unisense) were used in combination with a Unisense PA 2000 picoammeter, and pH and ion-selective sulfide needle (Microscale Measurements, The Netherlands) were deployed with a high-impedance microscale measurements millivolt meter (Microscale Measurements). The overlying water was gently stirred during the measurements. Because of the variations in mat surface topography and slight variations in the thickness of the different mat layers in smooth mats and thickness of the pustules, the depth of oxygen penetration and the observed maximum values of O_2_ and sulfide varies. Replicate measurements were made ca. 1 mm apart along a linear transect. This transect was marked and upon completion of electrode measurements, dissected for two-dimensional mapping of sulfate reduction activity (see below). Light measurements were performed using a LiCor LI 250 meter equipped with a SA190A quantum sensor.

Microbial metabolic rates were estimated using the light dark shift (for O_2_ production and consumption^72^,^73^). Briefly, the O_2_ concentration with depth was determined in the light. Following, the mats were incubated in the dark and O_2_ profiles measured every 10–15 min for ca. 1.5–2 h until profiles resembled dark profiles measured at 0400 h (i.e., depicting O_2_ diffusion into the mat). Consecutively, mats were exposed to natural sunlight and O_2_ profiles measured as before until the original light profile was approached. Oxygen consumption and production were calculated from the decrease, and increase minus decrease in [O_2_], respectively. Oxygenic photosynthesis produces oxygen, and aerobic respiration, chemolithotrophic sulfide oxidation and chemical reactions involving O_2_ are the key contributors to oxygen consumption. The 2D-distribution of sulfate-reducing activity was visualized using small strips (ca. 5 × 10 mm) of ref. 35 SO_4_^2^-labeled silver foil^74^ (ca. 0.1 mCi/mL Na_2_^35^ SO_4_; Perkin-Elmer, Waltham, MA). The principle of this technique is based on^35^ S-tracer use by sulfate reducing bacteria (SRB)^75^, where a vertical mat section was incubated on Ag foil coated with^35^ SO_4_^2^. Upon reduction by SRB, the metabolic product^35^ S^2−^ binds with the Ag foil surface and the radioactivity can be visualized using a radioactivity gel scanner^74^. The result is a quantitative high-resolution map of the distribution of SRB activity with depth. Freshly collected mat sections were cut vertically and placed on the Ag-foil strips. After 4 and 8 h of incubation at ca. 30 °C for the top and bottom of the core, respectively, the mat samples were removed and the remaining^35^ SO_4_^2^ rinsed off the foil using distilled water. The foils (containing^35^ S^2^ preserved as Ag^35^ S during SR) were kept in the dark and scanned using the BioRad Molecular Imager System GS-525 (Hercules, CA) to visualise a 2-D Ag^35^ S distribution. The result is a digital pixel map, in which darker pixels represent microzones of higher sulfate reducing activity. The spatial resolution of this technique is 140 × 140 μm.

Methane production was measured in samples upon return to the laboratory^56^,^60^. Based on *in situ* microelectrode O_2_ profiles, mats were separated in an oxic and anoxic part (0–3 and 3–20 mm for smooth mats and 0–6 and 6–10 mm for pustular mats, respectively). Slurries were prepared from entire layers of the mat, containing both organic (e.g., biomass and EPS) and inorganic (e.g., mineral) fractions. Individual slurries may comprise slightly different amounts of biomass, however identical biomass is not needed as the focus of these assays is on the overall differences in total community activity (methane production) between two different mat systems. Biomass differences may contribute to some of the differences observed and this is ecologically relevant. The samples were homogenised in 1:1 (v/v) mixture of 0.22 μm-filtered site water and mat and transferred to 18-ml crimp seal bottles (12 ml slurry and 6 ml headspace). Manipulations of mats and slurries were performed under a N_2_ atmosphere. The headspace of individual vials was flushed with N_2_ for 5 min at T = 0 except for the treatment in which the headspace was flushed with air daily. Triplicate samples were incubated in the dark at 25 °C on a shaker table (~60 rpm). The evolution of methane in the slurries was followed for 7–10 days by headspace analyses using gas chromatography with flame-ionisation detection (Shimadzu GC14A) and a PoraPak Q column and PeakSimple integration software^60^. In addition to appropriate controls, the following amendments were made to the slurries: trimethylamine (10 μM final concentration), H_2_/CO_2_ (75/675 μM), molybdate (28 mM) and BES (5 mM). In one treatment, the headspace was replaced daily by air.

### Nucleic acid extraction

Total community genomic DNA was extracted in duplicate from each microbial mat layer (10 layers from smooth mat, 5 from pustular mat) employing the MoBio PowerBiofilm DNA Isolation Kit (MO BIO Laboratories, Carlsbad, USA) according to the manufacturer’s instructions. A cross section of the mats indicating where they were layered is shown in Supplementary Figure 8. The concentrations and purity of extracted DNA were determined spectrometrically, and the quality checked by PCR amplification of archaeal 16S rRNA genes^12^,^13^,^28^.

### 16S SSU amplicon sequencing and analysis

Paired-End sequencing of archaeal 16S SSU genes from each mat layer in duplicate was performed using a MiSeq V3 2 × 300 bp kit on an Illumina MiSeq desktop sequencer^76^. Amplicons were produced using barcoded Arch-304F-Nex and Arch-915R-Nex primers. Amplicon primers were Arch-304F- (5′TCGTCGGCAGCGTCAGATGTGTATAAGAGACAGCCCTAHGGGGYGCASCA-3′), Arch-519R-(5′-GTCTCGTGGGCTCGGAGATGTGTATAAGAGACAGGWGCYCCCCCGYCAATTC-3′). MOTHUR software package version 1.33.0^77^ was employed. Quality control steps include removing sequence reads longer than 500 bp, removing homopolymers longer than 8 bp, and removing those containing ambiguous nucleotides. Sequences were then aligned (Needleman-Wunsch pairwise alignment) with the Silva bacteria database version 119^78^, and filtered to remove non-informative columns. Subsequently, the UCHIME function included in MOTHUR was employed to remove chimeric sequences in *de novo* mode^79^. After chimeras were removed, sequences were classified using default settings in MOTHUR against the Greengenes database^80^ (August 2013 released version). SSU sequences from each group were then randomly subsampled to 31,000 sequences (the fewest among the dataset) in order to normalise the sequence number for downstream statistical analyses. Subsequently, the sequences were clustered *de novo* into operational taxonomic units (OTU) at a genetic divergence level of 3%^81^ (or at 97% sequence identity). Taxonomic richness and diversity estimators were calculated using MOTHUR^77^.

### Statistical analyses

PRIMER 6 plus permanova packages were used for multivariate analyses, in which the relative abundance of each individual taxa was generated. The data was relative abundance of clustered OTUs at 97% cut-off for 16S rDNA based on archaea. The average relative abundance determined between duplicate layers is shown in Supplementary Table 2. Unless otherwise noted, abundance of OTUs were square root or natural log transformed in order to de-emphasize the large values; hence rare sequences of low abundance can also be taken into account. One-way Analysis of similarity test (ANOSIM) was performed to examine whether the microbial communities between both mat types were significantly different. PCA biplot analysis was performed in the Factomine package in R, to indicate how different archaeal groups putatively correlate to different depths in the mats. To further explore microbial stratification, statistical analysis of metagenomic profiles (STAMP) was undertaken^38^. Taxonomic groups from one layer were compared to the same taxonomic groups in all other layers, to identify which archaeal taxa in each layer were best discriminated significantly between other designated layers. Welch’s t-test was employed to reduce type I errors^82^, and Benjamini-Hochberg (i.e. false discovery rate) correction was applied as a multiple-hypothesis test correction. All quoted *P*-values represent corrected values (equating to *q*), with values <0.05 considered significant^83^. A filter was applied to remove taxa with a *q* value > 0.05.

### Data depositing

Sequences have been deposited in MG-RAST under accession numbers 4701089.3 to 4701857.3, and 4700514.3 to 4700879.3.

## Additional Information

**How to cite this article**: Wong, H. L. *et al*. Dynamics of archaea at fine spatial scales in Shark Bay mat microbiomes. *Sci. Rep.* **7**, 46160; doi: 10.1038/srep46160 (2017).

**Publisher's note:** Springer Nature remains neutral with regard to jurisdictional claims in published maps and institutional affiliations.

## Supplementary Material

Supplementary Information

## References

[b1] Méndez-García C. . Microbial stratification in low pH oxic and suboxic macroscopic growths along an acid mine drainage. ISME J. 8, 1259–1274 (2014). 24430486 10.1038/ismej.2013.242 PMC4030236

[b2] White R. A. III, Power I. M., Dipple G. M., Southam G. & Suttle C. A. Metagenomic analysis reveals that modern microbialites and polar microbial mats have similar taxonomic and functional potential. Front. Microbiol. 6, 966 (2015). 26441900 10.3389/fmicb.2015.00966 PMC4585152

[b3] Peeters K. . Heterotrophic bacterial diversity in aquatic microbial mat communities from Antartica. Polar. Biol. 35, 543–554 (2012).

[b4] White R. A. III . Metagenomic analysis suggests modern freshwater microbialites harbor a core distinct microbial community. Front. Microbiol. 6, 1531 (2016). 26903951 10.3389/fmicb.2015.01531 PMC4729913

[b5] Ley R. E. . Unexpected diversity and complexity of the Guerrero Negro hypersaline microbial mat. Appl. Envrion. Microbiol. 72 **(5)**, 3685–3695 (2006). 10.1128/AEM.72.5.3685-3695.2006 PMC1472358 16672518

[b6] Harris J. K. . Phylogenetic stratigraphy in the Guerrero Negro hypersaline microbial mat. ISME J. 7, 50–60 (2013). 22832344 10.1038/ismej.2012.79 PMC3526174

[b7] Schneider D., Arp G., Reimer A., Reitner J. & Daniel R. Phylogenetic analysis of a microbialite-forming microbial mat from a hypersaline lake of the Kiritimati Atoll, Central Pacific. Plos One 8 **(6)**, e66662 (2013). 23762495 10.1371/journal.pone.0066662 PMC3677903

[b8] Dupraz C., Visscher P. T., Baumgartner L. K. & Reid R. P. Microbe-mineral interactions: early carbonate precipitation in a hypersaline lake (Eleuthera Island, Bahamas). Sedimentol. 51, 745–765 (2004).

[b9] Baumgartner L. K. . Microbial Diversity in modern marine stromatolites, Highborne Cay, Bahamas. Environ. Microbiol. 11, 2710–2719 (2009). 19601956 10.1111/j.1462-2920.2009.01998.x

[b10] Dupraz C. D., Fowler A., Tobias C. & Visscher P. T. Microbialites of Storrs Lake, San Salvador, Bahamas: Knobs and Stromatolites Forming in an Organic Matrix. Geobiology 11, 527–548 (2013). 24118887 10.1111/gbi.12063

[b11] Glunk C. . Microbially-mediated carbonate precipitation in a hypersaline lake, Big Pond (Eleuthera, Bahamas). Sedimentology 58, 720–738 (2011).

[b12] Burns B. P., Goh F., Allen M. & Neila n. B. A. Microbial diversity of extant stromatolites in the hypersaline marine environment of Shark Bay, Australia. Environ. Microbiol. 6, 1096–1101 (2004). 15344935 10.1111/j.1462-2920.2004.00651.x

[b13] Ruvindy R., White R. A. III, Neilan B. A. & Burns B. P. Unravelling core microbial metabolisms in the hypersaline microbial mats of Shark Bay using high-throughput metagenomics. ISME J. 10 **(1)**, 183–196 (2016). 26023869 10.1038/ismej.2015.87 PMC4681862

[b14] Allen M. A., Goh F., Burns B. P. & Neilan B. A. Bacterial, archaeal and eukaryotic diversity of smooth and pustular microbial mat communities in the hypersaline lagoon of Shark Bay. Geobiology 7, 82–96 (2009). 19200148 10.1111/j.1472-4669.2008.00187.x

[b15] Papineau D., Walker J. J., Mojzsis S. J. & Pace N. R. Composition and structure of microbial communities from stromatolites of Hamelin Pool in Shark Bay, Western Australia. Appl. Environ. Microbiol. 71 **(8)**, 4822–4832 (2005). 16085880 10.1128/AEM.71.8.4822-4832.2005 PMC1183352

[b16] Jahnert R. J. & Collins L. B. Distribution and morphogenesis of subtidal microbial systems in Shark Bay, Australia. Mar. Geol. 303–306, 115–136 (2012).

[b17] Grotzinger J. P. & Knoll A. H. Stromatolites in Precambrian carbontates: Evolutionary mileposts of environmental dipsticks? Annu. Rev. Earth. Planet. Sci. 27, 313–358 (1999). 11543060 10.1146/annurev.earth.27.1.313

[b18] Playford P. E. Stromatolite research in western Australia. J. Royal. Soc. Western. Australia 62, 13–20 (1979).

[b19] Reid R. P., James N. P., MacIntyre I. H., Dupraz C. P. & Burne R. V. Shark Bay stromatolites: Microfabrics and reinterpretation of origins. Facies 49, 299–324 (2003).

[b20] Arp G., Reimer A. & Reitner J. Photosynthesis-induced biofilm calcification and calcium concentrations in Phanerozoic oceans. Science 292, 1701–1704 (2001). 11387471 10.1126/science.1057204

[b21] Arp G., Reimer A. & Reitner J. Calcification of cyanobacterial biofilms of alkaline salt lakes. Eur. J. Phycol. 34, 393–403 (1999).

[b22] Knauth L. P. Salinity history of Earth’s early ocean. Nature 395, 554 (1998). 11542867 10.1038/26879

[b23] Palmisano A. C., Summons R. E., Cronin S. E. & Marais D. J. Lipophilic pigments from cyanobacterial (Blue-green algal) and diatom mats in Hamelin Pool, Shark Bay, Western Australia. J. Phycol. 25, 633–661 (1989). 10.1111/j.0022-3646.1989.00655.x 11542174

[b24] Hoehler T. M., Bebout B. M. & Des Marais D. J. The role of microbial mats in the production of reduced gases on the early Earth. Nature 412, 324–327 (2001). 11460161 10.1038/35085554

[b25] Goh F. . Determining the specific microbial populations and their spatial distribution within the stromatolite ecosystem of Shark Bay. ISME J. 3, 383–396 (2009). 19092864 10.1038/ismej.2008.114

[b26] Van Gemerden H. Microbial mats: a joint venture. Mar. Geol. 113, 3–25 (1993).

[b27] Visscher P. T., Beukema J. & Van Gemerden H. In situ characterization of sediments: measurements of oxygen and sulphide profiles. Limnol. Oceanogr. 36, 1476–1480 (1991).

[b28] Wong H. L., Smith D. L., Visscher P. T. & Burns B. P. Niche differentiation of bacterial communities at a millimiter scale in Shark Bay microbial mats. Sci Rep. 5 (2015). 10.1038/srep15607 PMC4620479 26499760

[b29] Dupraz C. & Visscher P. T. Microbial lithification in modern marine stromatolites and hypersaline mats. Trends. Microbiol. 13 **(9)**, 429–438 (2005). 16087339 10.1016/j.tim.2005.07.008

[b30] Kunin V. . Millimeter-scale genetic gradients and community-level molecular convergence in a hypersaline microbial mat. Mol. Syst. Biol. 4, 198–203 (2008). 18523433 10.1038/msb.2008.35 PMC2483411

[b31] Burow L. C. . Anoxic carbon flux in photosynthetic microbial mats as revealed by metatranscriptomics. ISME J. 7, 817–829 (2013). 23190731 10.1038/ismej.2012.150 PMC3603402

[b32] Mobberley J. M. . Inner workings of thrombolites: spatial gradients of metabolic activity as revealed by metatranscriptome profiling. Sci Reps. 5 (2015). 10.1038/srep12601 PMC4515876 26213359

[b33] Buckley D. H., Baumgartner L. K. & Visscher P. T. Vertical distribution of methane metabolism in microbial mats of the Great Sippewissett Salt Marsh. Environ. Microbiol. 10 **(4)**, 967–977 (2008). 18218028 10.1111/j.1462-2920.2007.01517.x

[b34] Goh F. . *Halococcus hamelinensis* sp. nov., a novel halophilic archaeon isolated from stromatolites in Shark Bay, Australia. Int. J. Syst. Evol. Microbiol. 56, 1323–1329 (2006). 16738110 10.1099/ijs.0.64180-0

[b35] Allen M. A. . *Haloferax elongans* sp. nov. and *Haloferax mucosum* sp. nov., isolated from microbial mats from Hamelin Pool, Shark Bay. Int. J. Syst. Evol. Microbiol. 58, 798–802 (2008). 18398172 10.1099/ijs.0.65360-0

[b36] Rinke C. . Insights into the phylogeny and coding potential of microbial dark matter. Nature 499, 431–437 (2013). 23851394 10.1038/nature12352

[b37] Hedlund B. P., Dodsworth J. A., Murugapiran S. K., Rinke C. & Woyke T. Impact of single-cell genomics and metagenomics on the emerging view of extremophile “microbial dark matter”. Extrememophiles 18 **(5)**, 865–875 (2014). 10.1007/s00792-014-0664-7 25113821

[b38] Parks D. H., Tyson G. W., Hugenholtz P. & Beiko R. G. STAMP: Statistical analysis of taxonomic and functional profiles. Bioinformatics 30 **(21)**, 3123–3124 (2014). 25061070 10.1093/bioinformatics/btu494 PMC4609014

[b39] Goh F., Barrow K. D., Burns B. P. & Neilan B. A. Identification and regulation of novel compatible solutes from hypersaline stromatolite-associated cyanobacteria. Arch. Microbiol. 192, 1031–1038 (2010). 20936259 10.1007/s00203-010-0634-0

[b40] Goh F., Jeon Y.-J., Barrow K. D., Neilan B. A. & Burns B. P. Osmoadaptive strategies of the archaeon *Halococcus hamelinensis* isolated from a hypersaline stromatolite environment. Astrobiology 11, 529–536 (2011). 21810017 10.1089/ast.2010.0591

[b41] Jeffries T. C. . Increases in the abundance of microbial genes encoding halotolerance and photosynthesis along a sediment salinity gradient. Biogeosciences 9, 815–825 (2012).

[b42] Diaz M. R., Visscher P. T. & Taylor B. F. Metabolism of dimethylsulfoniopropionate and glycine betaine by a marine bacterium. FEMS Microbiol. Lett. 96, 61–66 (1992).

[b43] Oren A. Formation and breakdown of glycine betaine and trimethylamine in hypersaline environments. Antonie van Leeuwenhoek 58, 291–298 (1990). 2082817 10.1007/BF00399342

[b44] Hedlund B. P., Dodsworth J. A., Cole J. K. & Panosyan H. H. An integrated study reveals diverse methanogens, Thaumarchaeota, and yet-uncultivated archaeal lineages in Armenian hot springs. Antonie van Leeuwenhoek 104, 71–82 (2013). 23632917 10.1007/s10482-013-9927-z

[b45] Daims H. . Complete nitrification by Nitrospira bacteria. Nature 528 **(7583)**, 504–509 (2015). 26610024 10.1038/nature16461 PMC5152751

[b46] Rouvière P., Mandelco L., Winker S. & Woese C. R. A detailed phylogeny for the methanomicrobiales. Syst. Appl. Microbiol. 15, 363–371 (1992). 11540078 10.1016/S0723-2020(11)80209-2

[b47] Smith J. M., Green S. J., Kelley C. A., Prufert-Bebout L. & Bebout B. M. Shifts in methanogen community structure and function associated with long-term manipulation of sulfate and salinity in a hypersaline microbial mat. Environ. Microbiol. 10 **(2)**, 386–394 (2008). 18177370 10.1111/j.1462-2920.2007.01459.x

[b48] King G. M. Methanogenesis from methylated amines in a hypersaline algal mat. Appl. Environ. Microbiol. 54, 130–136 (1988). 16347519 10.1128/aem.54.1.130-136.1988 PMC202409

[b49] Lazar C. S., Parkes R. J., Cragg B. A., L’Haridon S. & Toffin L. Methanogenic diversity and activity in hypersaline sediments of the centre of the Napoli mud volcano, Eastern Mediterranean Sea. Environ. Microbiol. 13 **(8)**, 2078–2091 (2011). 21382146 10.1111/j.1462-2920.2011.02425.x

[b50] García-Maldonado J. Q., Bebout B. M., Celis L. B. & López-Cortés A. Phylogenetic diversity of methyl-coenzyme M reductase (mcrA) gene and methanogenesis from trimethylamine in hypersaline environments. Int. Microbiol. 15, 33–41 (2012). 22837150 10.2436/20.1501.01.156

[b51] Oren A. Diversity of halophilic microorganisms: Environments, phylogeny, physiology, and applications. J. Ind. Microbiol. Biotechnol. 28, 56–63 (2002). 11938472 10.1038/sj/jim/7000176

[b52] Oremland R. S. & Polcin S. Methanogenesis and sulfate reduction – competitive and noncompetitive substrates in estuarine sediments. Appl. Environ. Microbiol. 44, 1270–1276 (1982). 16346144 10.1128/aem.44.6.1270-1276.1982 PMC242184

[b53] King G. M., Klug M. J. & Lovley D. R. Metabolism of acetate, methanol, and methylated amines in intertidal sediments of Lowes Cove, Mainet. Appl. Environ. Microbiol. 45, 1848–1853 (1983). 16346317 10.1128/aem.45.6.1848-1853.1983 PMC242548

[b54] King G. M. Metabolism of trimethylamine, choline, and glycine betaine by sulfate-reducing and methanogenic bacteria in marine-sediments. Appl. Environ. Microbiol. 48, 719–725 (1984). 16346640 10.1128/aem.48.4.719-725.1984 PMC241601

[b55] Kiene R. P., Oremland R. S., Catena A., Miller L. G. & Capone D. G. Metabolism of reduced methylated sulphur-compounds in anaerobic sediments and by a pure culture of an estuarine methanogen. Appl. Environ. Microbiol. 52, 1037–1045 (1986). 16347202 10.1128/aem.52.5.1037-1045.1986 PMC239170

[b56] Visscher P. T. & Van Gemerden H. Production and consumption of dimethylsulfoniopropionate in marine microbial mats. Appl. Environ. Microbiol. 57 **(11)**, 3237–3242 (1991). 16348586 10.1128/aem.57.11.3237-3242.1991 PMC183954

[b57] Visscher P. T. . Biogeochemistry of carbon cycling in hypersaline mats: linking the present to the past through biosignatures, In Microbioal Mats. Springer, Netherlands, pp 443–468 (2010).

[b58] Pagès A. . Diel fluctuations in solute distributions and biogeochemical cycling in a hypersaline microbial mat from Shark Bay, WA. Mar. Chem. 167, 102–112 (2014).

[b59] Watkins A. J., Roussel E. G., Parkes R. J. & Sass H. Glycine betaine as a direct substrate for methanogens (Methanococcoides spp.). Appl. Environ. Microb. 80 **(1)**, 289–293 (2014). 10.1128/AEM.03076-13 PMC3911008 24162571

[b60] Visscher P. T. . Dimethyl Sulfide and Methanethiol Formation in Microbial Mats: Potential Pathways for Biogenic Signatures. Environ. Microbiol. 5, 296–308 (2003). 12662177 10.1046/j.1462-2920.2003.00418.x

[b61] Burow L. C. . Identification of Desulfobacterales as primary hydrogenotrophs in a complex microbial mat community. Geobiology 12, 221–230 (2014). 24730641 10.1111/gbi.12080

[b62] Skyring G. W., Lynch R. M. & Smith G. D. Quantitative relationships between carbon, hydrogen and sulfur metabolism in cyanobacterial mats. In Cohen Y., Rosenberg E. (ed). Microbial Mats: Physiological Ecology of Benthic Microbial Communities American Society for Microbiology, Washington, DC, pp 170–179 (1989).

[b63] Ferrer M. . Taxonomic and functional metagenomics profiling of the microbial community in the anoxic sediment of a sub-saline shallow lake (Laguna de Carrizo, Central Spain). Microb. Ecol. 62 **(4)**, 824–837 (2011). 21735153 10.1007/s00248-011-9903-y

[b64] Wemheuer B., Wemheuer F. & Daniel R. RNA-based assessment of diversity and composition of active archaeal communities in the German Bight. Archaea Article ID 695826 (2012). 10.1155/2012/695826 PMC3502831 23197941

[b65] Petrisor A. . Changing microspatial patterns of sulfate-reducing microorganisms (SRM) during cycling of marine stromatolite mats. Int. J. Mol. Sci. 15, 850–877 (2014). 24413754 10.3390/ijms15010850 PMC3907843

[b66] Decho A. W., Norman R. S. & Visscher P. T. Quorum sensing in natural environments: emerging views from microbial mats. Trends. Microbiol. 18, 73–80 (2010). 20060299 10.1016/j.tim.2009.12.008

[b67] Burns B. P. . Modern analogues and the early history of microbial life. Precambrian. Res. 173, 10–18 (2009).

[b68] García-Maldonado J. Q., Bebout B. M., Everroad R. C. & López-Cortés A. Evidence of novel phylogenetic lineages of methanogenic archaea from hypersaline microbial mats. Microb. Ecol. 69, 106–117 (2015). 25108574 10.1007/s00248-014-0473-7

[b69] Bapteste É., Brochier C. & Boucher Y. Higher-level classification of the Archaea: evolution of methanogenesis and methanogens. Archaea 1, 353–363 (2005). 15876569 10.1155/2005/859728 PMC2685549

[b70] Valdivieso-Ojeda J. A., Huerta-Diaz M. A. & Delgadillo-Hinojosa F. High enrichment of molybdenum in hypersaline microbial mats of Guerrero Negro, Baja California Sur, Mexico. Chem. Geol. 363, 341–354 (2014).

[b71] Boyd E. S. . A late methanogen origin for molybdenum-dependent nitrogenase. Geobiology 9, 221–232 (2011). 21504537 10.1111/j.1472-4669.2011.00278.x

[b72] Revsbech N. P., Jørgensen B. B. & Brix O. Primary production of microalgae in sediments measured by oxygen microprofile, H^14^CO_3_^−^ fixation, and oxygen exchange methods. Limnol. Oceanogr. 26,717–730 (1981).

[b73] Revsbech N. P. & Jørgensen B. B. Photosynthesis of benthic microflora measured with high spatial resolution by the oxygen microprofile method: capabilities and limitations of the method. Limnol. Oceanogr. 28,749–756 (1983).

[b74] Visscher P. T., Reid R. P. & Bebout B. M. Microscale observations of sulfate reduction: evidence of microbial activity forming lithified micritic laminae in modern marine stromatolites. Geology 28, 919–922 (2000).

[b75] Jørgensen B. B. A comparison of methods for the quantification of bacterial sulfate reduction in coastal marine sediments. I. Measurement with radiotracer techniques. Geomicrobiol. J. 1, 11–28 (1978).

[b76] Caporaso J. G. . Ultra-high-throughput microbial community analysis on the Illumina HiSeq and MiSeq platforms. ISME J. 6, 1621–1624 (2012). 22402401 10.1038/ismej.2012.8 PMC3400413

[b77] Schloss P. D. . Introducing mothur: open-source, platform-independent, community-supported software for describeing and comparing microbial communities. Appl. Environ. Microbiol. 75, 7537–7541 (2009). 19801464 10.1128/AEM.01541-09 PMC2786419

[b78] Quast C. . The SILVA ribosomal RNA gene database project: improved data processing and web-based tools. Nucl. Acid. Res. 41, 590–596 (2013). 10.1093/nar/gks1219 PMC3531112 23193283

[b79] Egar R. C., Haas B. J., Clemente J. C., Quince C. & Knight R. UCHIME improves sensitivity and speed of chimera detection. Bioinformatics 27, 2194–2200 (2011). 21700674 10.1093/bioinformatics/btr381 PMC3150044

[b80] DeSantis T. Z. . Greengenes, a chimera-checked 16S rRNA gene database and workbench compatible with ARB. Appl. Environ. Microbiol. 72, 5069–5072 (2006). 16820507 10.1128/AEM.03006-05 PMC1489311

[b81] Kozich J. J., Westcott S. L., Baxter N. T., Highlander S. K. & Schloss P. D. Development of a dual-index sequencing strategy and curation pipeline for analysing amplicon sequence data on the MiSeq Illumina sequencing platform. Appl. Environ. Microbiol. 79, 5112–5120 (2013). 23793624 10.1128/AEM.01043-13 PMC3753973

[b82] Ruxton G. D. The unequal variance t-test is an underused alternative to Student’s t-test and the Mann-Whitney u test. Behav. Ecol. 17, 688–690 (2006).

[b83] Clarke K. R. & Ainsworth M. A method of linking multivariate community structure to environmental variables. Mar. Ecol. Prog. Ser. 92, 205–219 (1993).

