# Supplementary material for: Dynamics of archaea at fine spatial scales in Shark Bay mat microbiomes
Source: Sci Rep. 2017 Apr 11;7:46160. doi: 10.1038/srep46160 (PMC5387419; doi:10.1038/srep46160)
Supplement: Supplementary Information [file srep46160-s1.pdf]

Dynamics of archaea at fine spatial scales in Shark Bay mat microbiomes¶

Hon Lun Wong[1,3*], Pieter T. Visscher[2,3*], Richard Allen White III[4*], Daniela-Lee Smith[1], Molly M. Patterson[2], Brendan P. Burns[1,3]

[1]School of Biotechnology and Biomolecular Sciences, The University of New South Wales, Sydney, Australia

[2]Department of Marine Sciences, University of Connecticut, USA

[3]Australian Centre for Astrobiology, University of New South Wales Sydney, Australia

[4]Earth and Biological Sciences Directorate, Pacific Northwest National Laboratory, Richland,
Washington 99352, USA

Supplementary information of figures/tables accompanying SREP-16-45815.

Supplementary Figure 1. Rarefaction curves of smooth and pustular mats layers. The bottom layer of pustular mats (PL5, 8 – 10 mm) has the highest number of different OTUs, followed by the bottom layer of smooth mats (SL10, 18 – 20 mm) and the 6th layer of smooth mats (SL6, 10 – 12 mm). PL1 to PL5 refer to pustular mat layers 1 - 5, while SL1 – SL10 refer to smooth mat layers 1 – 10.

Supplementary Figure 2. Abundance of prominent classes and taxonomic subgroups by depth in smooth mats. A. Parvarchaeota, **B**. Thermoplasmata, **C**. Methanogens, **D**. Others, including Marine Benthic Group B (MBGB) (Under phylum Crenarachaeota in Greengenes database, while under Thaumarchaeota in Silva database), Halobacteria, Thermoprotei, Thermococci and Miscellaneous Crenarchaeota Group (MCG).

Supplementary Figure 3. STAMP analysis of taxonomy enriched or depleted between the designated layers and all other layers in smooth mats. Surface group compared to the other layers (sub-surface, anoxic, bottom layers), showing the surface of smooth mats is characterised by Parvarchaeota order YLA114 and Thermoplasmata lineage CCA47.

Supplementary Figure 4. STAMP analysis of taxonomy enriched or depleted between the designated layers and all other layers in smooth mats. Subsurface compared to all other layers (surface, anoxic, bottom layers), showing subsurface is represented by class Marine Benthic Group B (MBGB).

Supplementary Figure 5. STAMP analysis of taxonomy enriched or depleted between the designated layers and all other layers in smooth mats (obtained from cluster analysis in Figure 2). Bottom layer compared to all other layers (surface, sub-surface, anoxic), showing bottom is characterised mainly by Parvarchaeota order WCHD3-30, various methylotrophic methanogens (Methanobacteriales, Methanococcales) and Thermoplasmata lineage DHVEG-1.

Supplementary Figure 6. Abundance of prominent classes and taxonomic subgroups by depth in pustular mats. **A**. Halobacteria, **B**. Parvarchaeota and Thaumarchaeota. Vertical depth of 10 mm, dissected into five two mm-layers. Lines indicate the line of sectioning.

Supplementary Figure 7. Measurements of water depth, salinity, temperature and light intensity in Shark Bay at the time of sampling. Measurements were taken during the transition from high tide to low tide in smooth (A) and pustular (B) microbial mats in Nilemah, Shark Bay.

Supplementary Figure 8. Vertical cross section of Shark Bay microbial mats indicating layers analysed. A. Smooth mat sample of 2 cm vertical depth, dissected into ten 2 mm-layers. **B**. Pustular mat sample of 1 cm vertical depth, dissected into five 2 mm-layers. Lines indicate the line of sectioning.

Supplementary Table 1. Archaeal 16S rRNA gene diversity analyses of smooth and pustular mats in Shark Bay.

Supplementary Table 2. Relative abundance of archaeal classes in smooth and pustular mats.

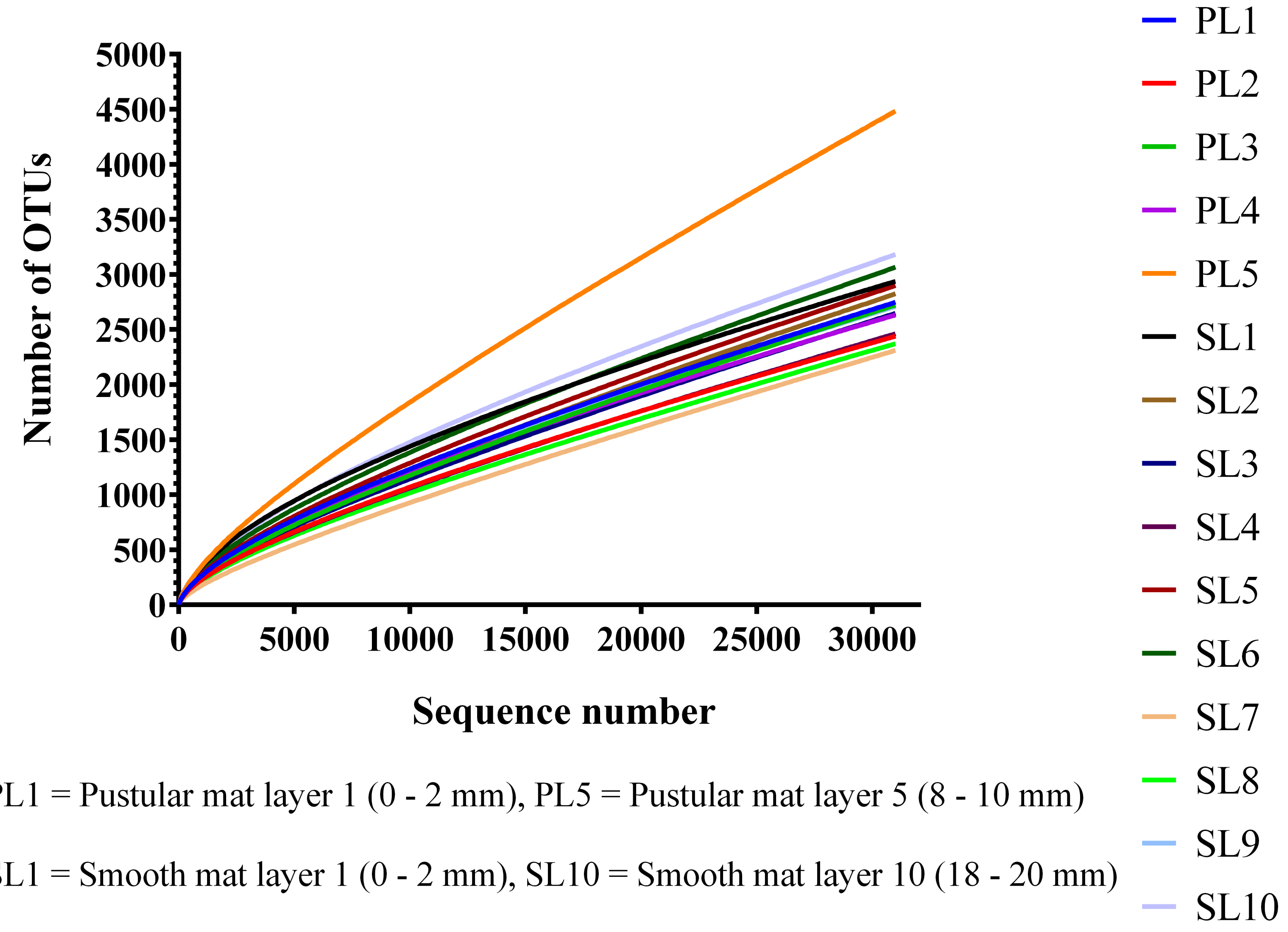

PL1 = Pustular mat layer 1 (0 - 2 mm), PL5 = Pustular mat layer 5 (8 - 10 mm)

SL1 = Smooth mat layer 1 (0 - 2 mm), SL10 = Smooth mat layer 10 (18 - 20 mm)

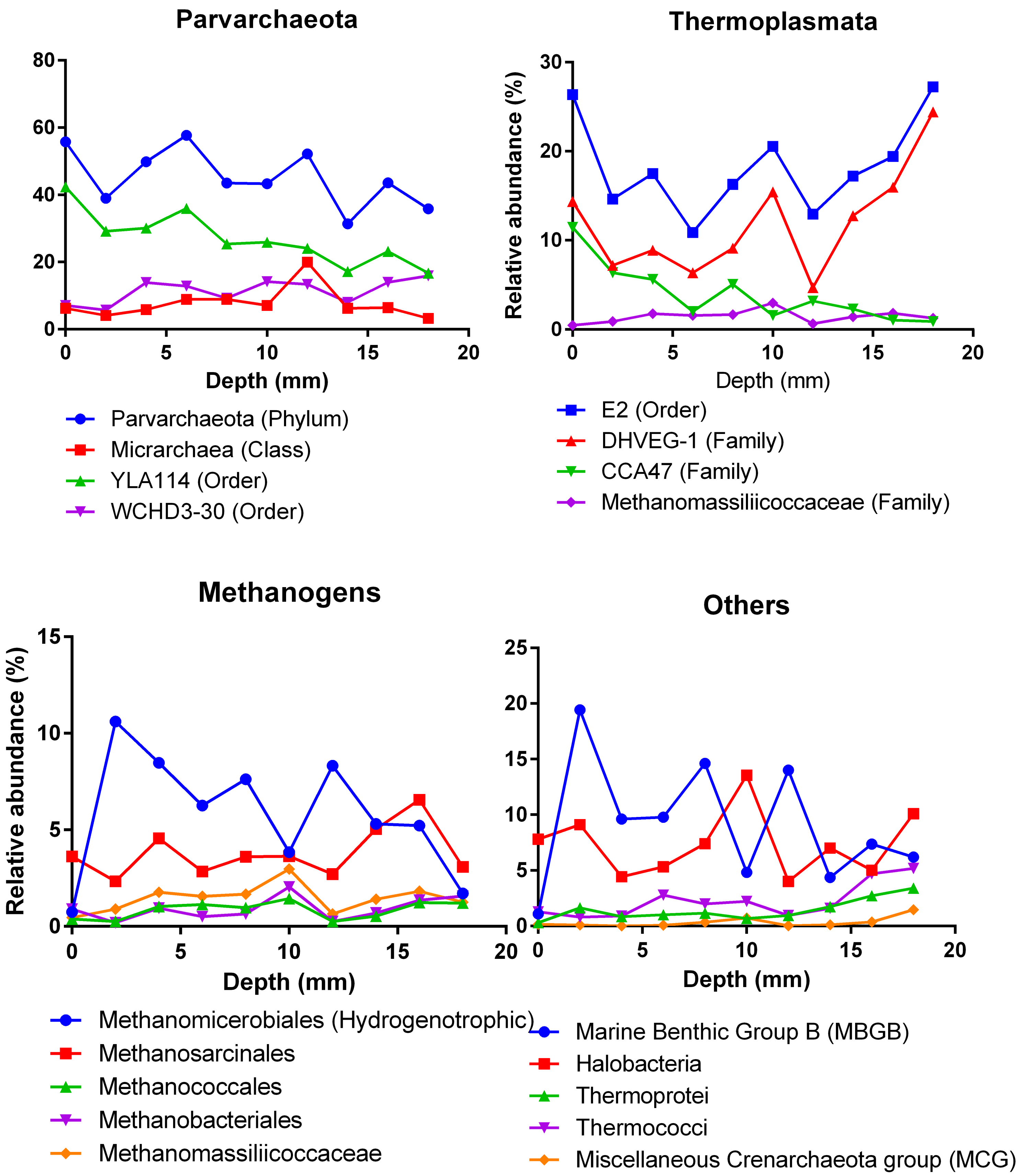

Parvarchaeota
80
60
40
20
0
0
5
10
15
20
Depth (mm)
Parvarchaeota (Phylum)
Micrarchaea (Class)
YLA114 (Order)
WCHD3-30 (Order)
Thermoplasmata
Relative abundance (%)
30
20
10
0
Depth (mm)
E2 (Order)
DHVEG-1 (Family)
CCA47 (Family)
Methanomassiliicoccaceae (Family)
Methanogens
Relative abundance (%)
15
10
5
Depth (mm)
Methanomicerobiales (Hydrogenotrophic)
Methanosarcinales
Methanococcales
Methanobacteriales
Methanomassiliicoccaceae
Others
25
Depth (mm)
Marine Benthic Group B (MBGB)
Halobacteria
Thermoprotei
Thermococci
Miscellaneous Crenarchaeota group (MCG)

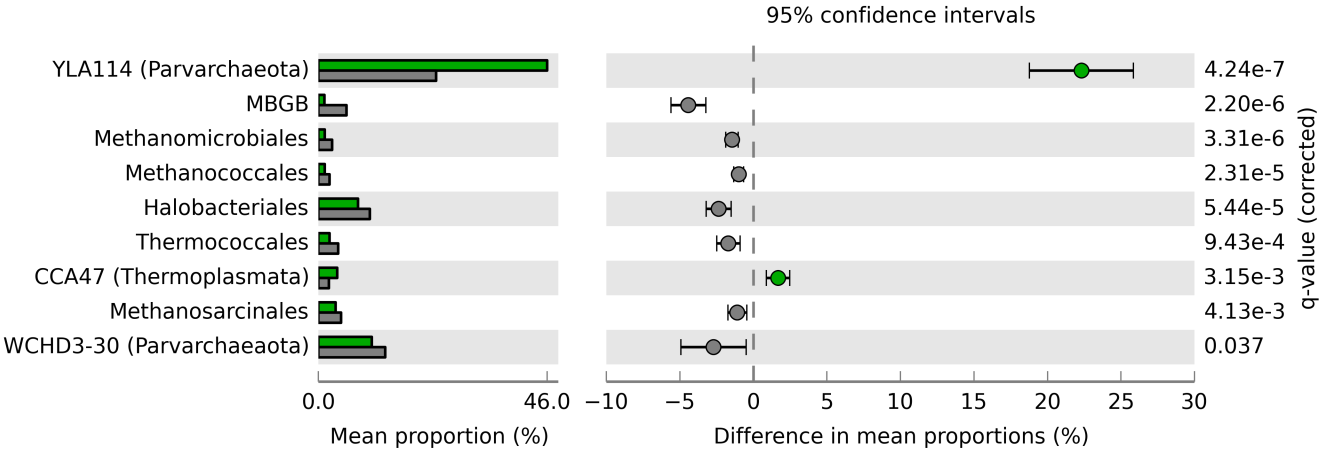
95% confidence intervals
YLA114 (Parvarchaeota)
MBGB
Methanomicrobiales
Methanococcales
Halobacteriales
Thermococcales
CCA47 (Thermoplasmata)
Methanosarcinales
WCHD3-30 (Parvarchaeaota)
0.0
46.0
Mean proportion (%)
−10
−5
0
5
10
15
20
25
30
Difference in mean proportions (%)
4.24e-7
2.20e-6
3.31e-6
2.31e-5
5.44e-5
9.43e-4
3.15e-3
4.13e-3
0.037
q-value (corrected)

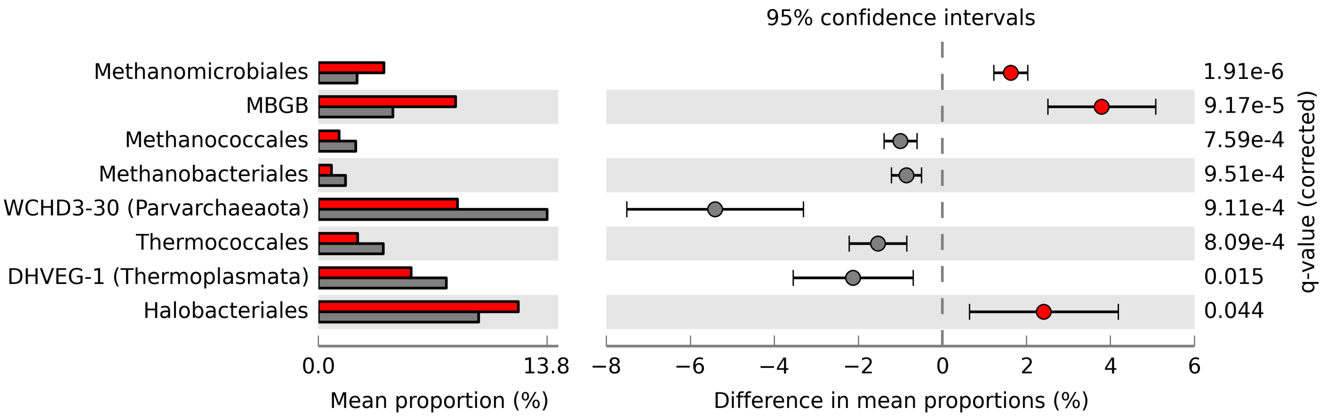
95% confidence intervals
Methanomicrobiales
MBGB
Methanococcales
Methanobacteriales
WCHD3-30 (Parvarchaeaota)
Thermococcales
DHVEG-1 (Thermoplasmata)
Halobacteriales
0.0
13.8
Mean proportion (%)
−8
−6
−4
−2
0
2
4
6
Difference in mean proportions (%)
1.91e-6
9.17e-5
7.59e-4
9.51e-4
9.11e-4
8.09e-4
0.015
0.044
q-value (corrected)

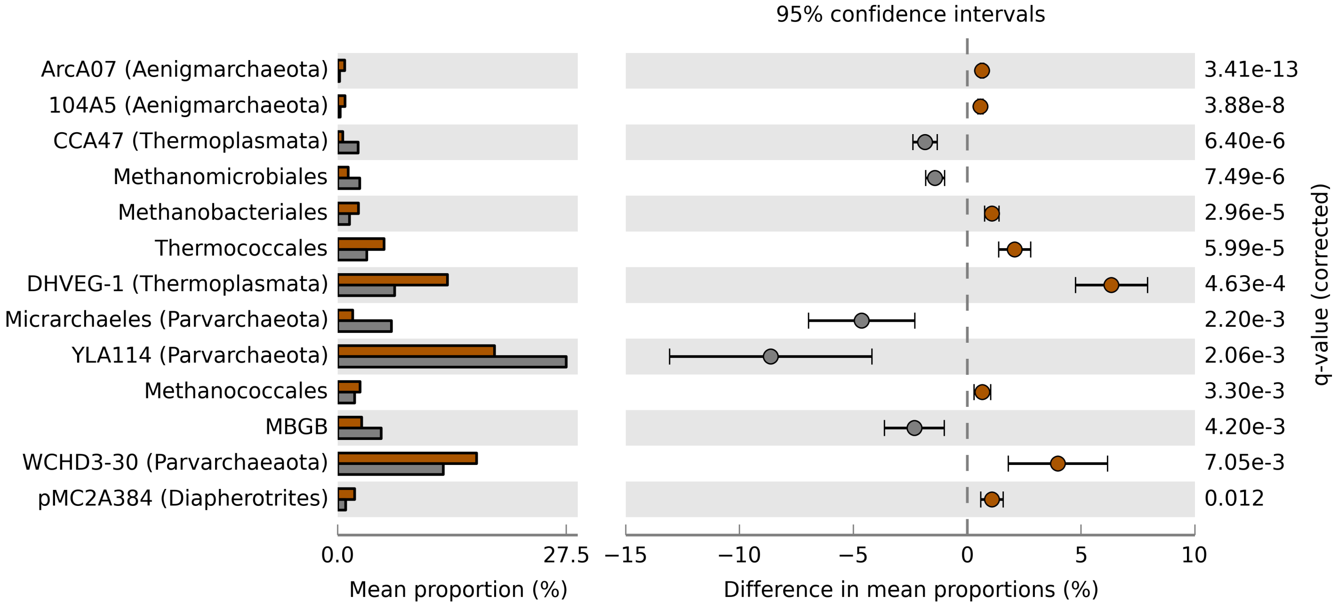
95% confidence intervals
ArcA07 (Aenigmarchaeota)
104A5 (Aenigmarchaeota)
CCA47 (Thermoplasmata)
Methanomicrobiales
Methanobacteriales
Thermococcales
DHVEG-1 (Thermoplasmata)
Micrarchaeles (Parvarchaeota)
YLA114 (Parvarchaeota)
Methanococcales
MBGB
WCHD3-30 (Parvarchaeaota)
pMC2A384 (Diapherotrites)
3.41e-13
3.88e-8
6.40e-6
7.49e-6
2.96e-5
5.99e-5
4.63e-4
2.20e-3
2.06e-3
3.30e-3
4.20e-3
7.05e-3
0.012
q-value (corrected)
0.0
27.5
Mean proportion (%)
−15
−10
−5
0
5
10
Difference in mean proportions (%)

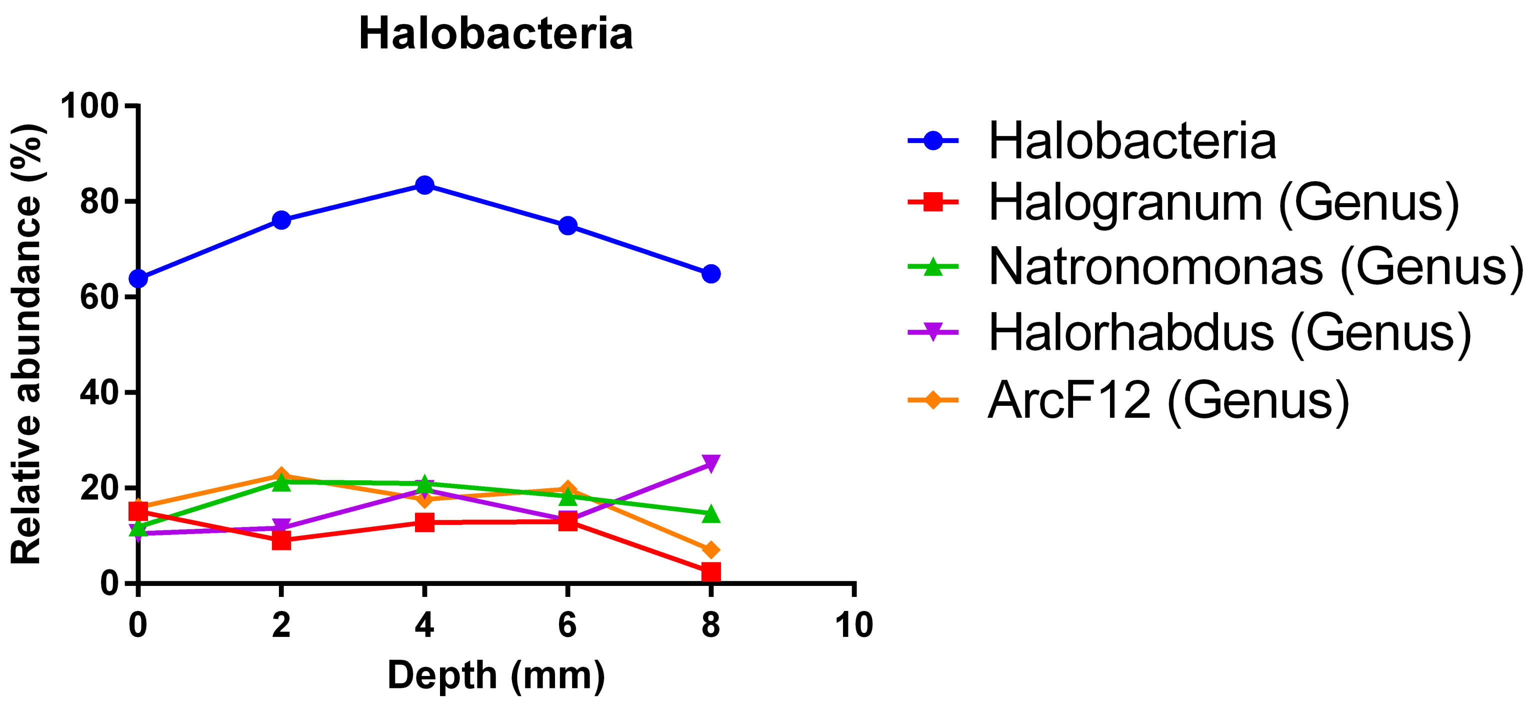
Halobacteria
Relative abundance (%)
100
80
60
40
20
0
0
2
4
6
8
10
Depth (mm)
Halobacteria
Halogranum (Genus)
Natronomonas (Genus)
Halorhabdus (Genus)
ArcF12 (Genus)

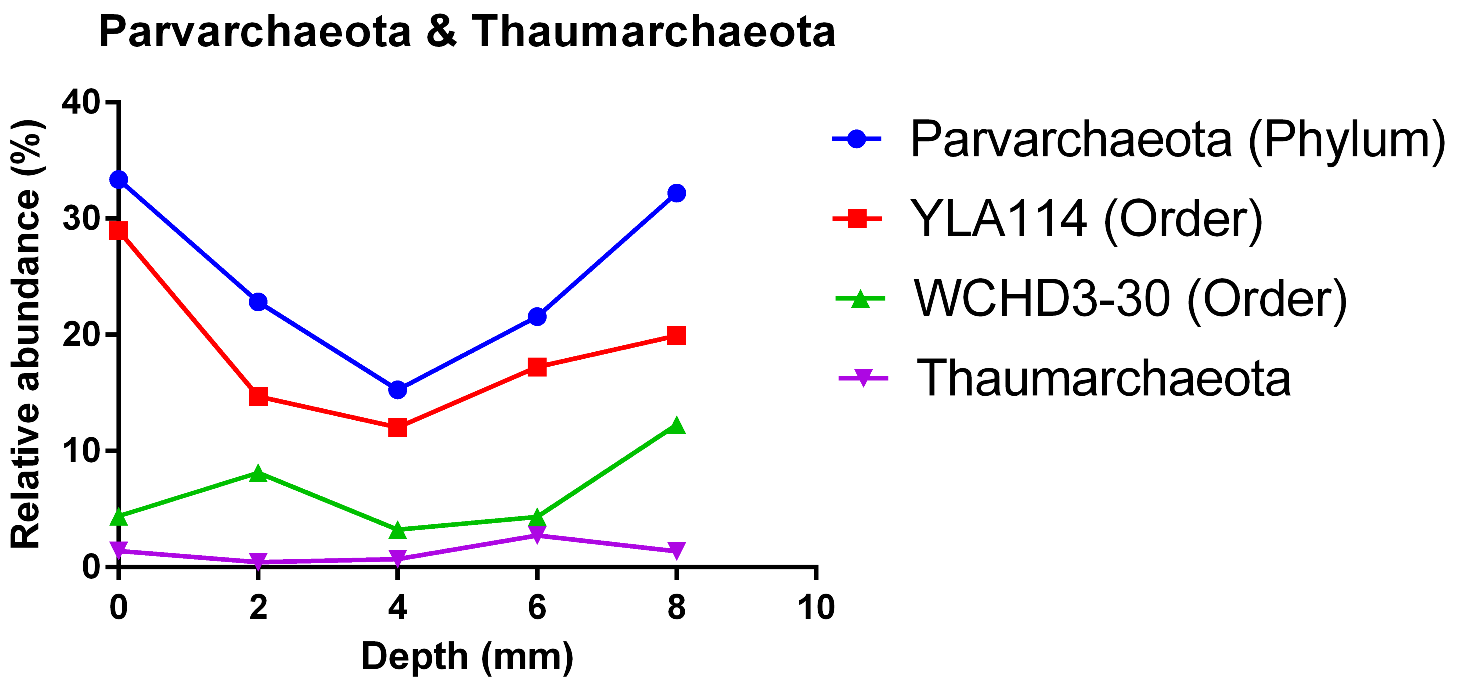
Parvarchaeota & Thaumarchaeota
Relative abundance (%)
40
30
20
10
0
0
2
4
6
8
10
Depth (mm)
Parvarchaeota (Phylum)
YLA114 (Order)
WCHD3-30 (Order)
Thaumarchaeota

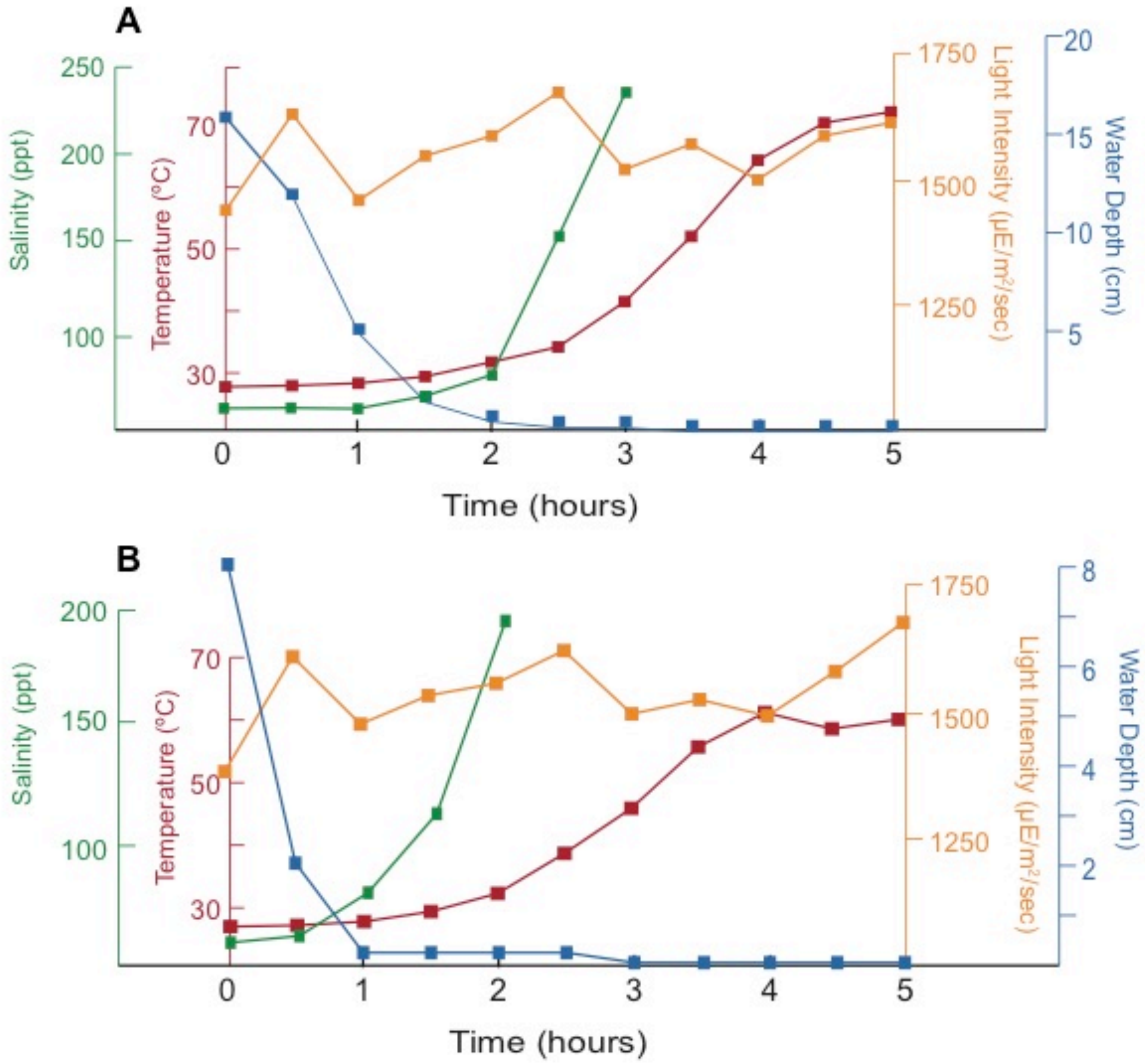
A
Salinity (ppt)
250
200
150
100
Temperature (°C)
70
50
30
Light Intensity (µE/m²/sec)
1750
1500
1250
Water Depth (cm)
20
15
10
5
0
1
2
3
4
5
Time (hours)
B
Salinity (ppt)
200
150
100
Temperature (°C)
70
50
30
Light Intensity (µE/m²/sec)
1750
1500
1250
Water Depth (cm)
8
6
4
2
0
1
2
3
4
5
Time (hours)

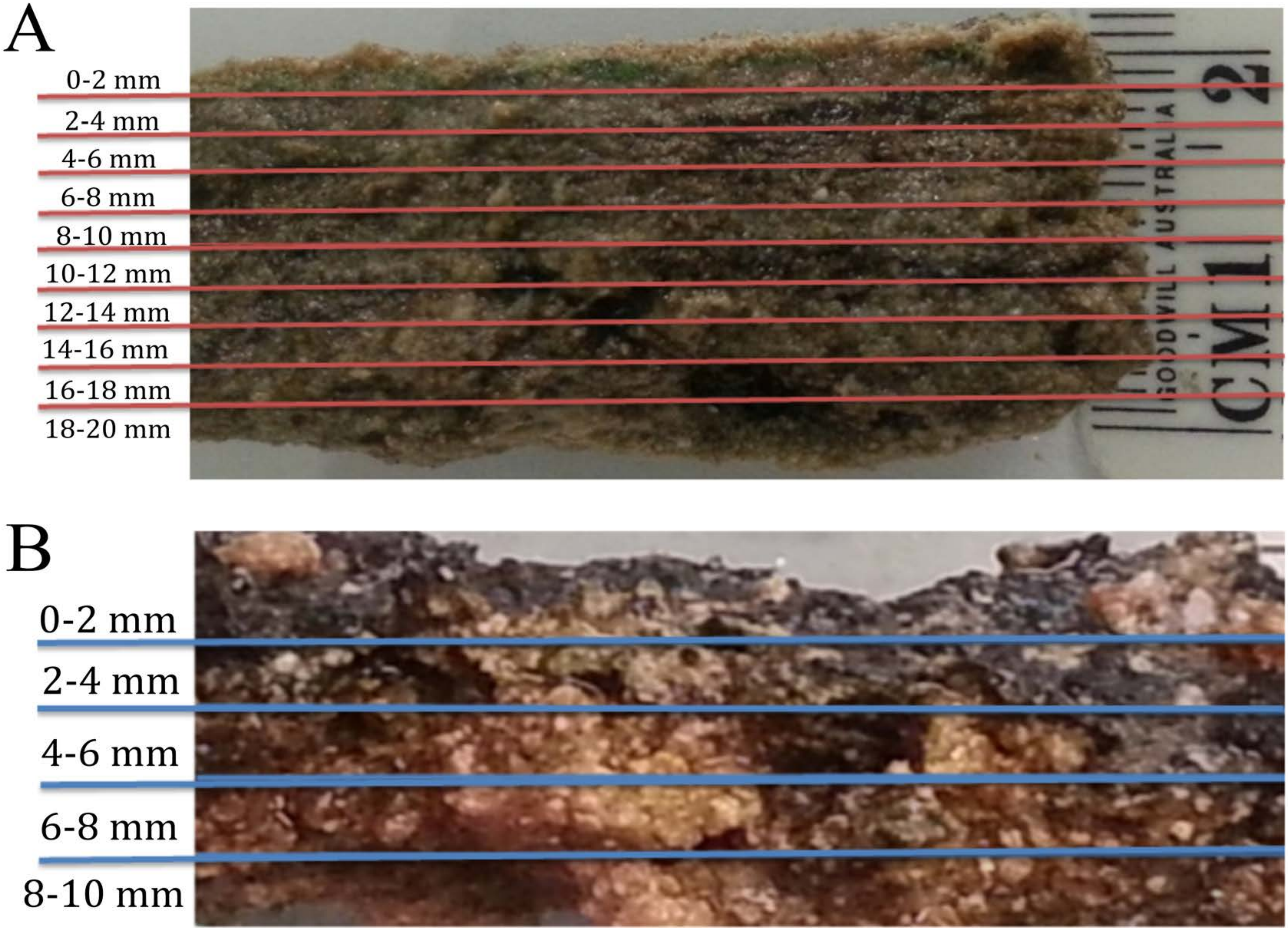
A
0-2 mm
2-4 mm
4-6 mm
6-8 mm
8-10 mm
10-12 mm
12-14 mm
14-16 mm
16-18 mm
18-20 mm
CM 1 2
GODWILL AUSTRALIA
B
0-2 mm
2-4 mm
4-6 mm
6-8 mm
8-10 mm

Supplementary Table 1. Archaeal 16S rRNA gene diversity analyses* of smooth and pustular mats in Shark Bay.

Mat layers	Depth	No. of OTUs	Chao[a]	Shannon[b]	Coverage[c]
PL1	0 - 2 mm	2748.3	14952	4.805	93.45%
PL2	2 - 4 mm	2441	13653	4.506	94.04%
PL3	4 -6 mm	2723.3	15036	4.678	93.24%
PL4	6 - 8 mm	2633.5	12527	4.829	93.82%
PL5	8 - 10 mm	4484	28993	5.667	88.31%
SL1	0 - 2 mm	2938.3	11128	5.418	93.73%
SL2	2 - 4 mm	2825.3	15838	4.618	92.97%
SL3	4 -6 mm	2648	15979	4.807	93.38%
SL4	6 - 8 mm	2462.5	14880	4.837	93.84%
SL5	8 - 10 mm	2900.7	14704	5.056	93.03%
SL6	10 - 12 mm	3067	14433	5.435	92.72%
SL7	12 - 14 mm	2311.5	19186	3.982	93.79%
SL8	14 - 16 mm	2372	15250	4.503	93.99%
SL9	16 - 18 mm	2710.5	14445	5.152	93.51%
SL10	18 - 20 mm	3182	14474	5.671	92.74%

*Calculated at 3% genetic divergence

[a]Chao index measures the species richness (absolute number of species)

[b]Shannon index measures the species diversity (relative species abundance and species evenness)

[c]Good's Coverage measures how well the sample represents the larger environment

Supplementary Table 2. Relative abundance of archaeal classes in smooth and pustular mats[a]

	Halobacteria	MBGB	Micrarchaea	Thermoplasmata	YLA114[b]	WCHD3-30[b]	Methanomicrobia	*Nitrosopumilus*	Others[c]
Smooth mat (0-20 mm)[d]	6.91%	9.74%	7.84%	19.99%	27.66%	11.00%	10.17%	N/A	6.67%
S1 (0-2 mm)[e]	7.80%	1.10%	6.26%	27.23%	42.39%	7.09%	4.38%	N/A	3.75%
S2 (2-4 mm)[e]	9.11%	19.42%	4.11%	16.44%	29.14%	5.68%	12.95%	N/A	3.16%
S3 (4-6 mm)[e]	4.42%	9.61%	5.81%	19.15%	30.13%	13.87%	13.06%	N/A	3.94%
S4 (6-8 mm)[e]	5.32%	9.79%	8.93%	12.00%	35.96%	12.80%	9.11%	N/A	6.10%
S5 (8-10 mm)[e]	7.41%	14.62%	8.92%	17.70%	25.35%	9.25%	11.26%	N/A	5.51%
S6 (10-12 mm)[e]	7.95%	6.66%	6.37%	22.05%	25.89%	14.15%	7.55%	N/A	9.37%
S7 (12-14 mm)[e]	4.00%	17.56%	19.98%	10.62%	24.23%	7.94%	12.96%	N/A	2.70%
S8 (14-16 mm)[e]	8.00%	5.09%	8.43%	24.83%	23.68%	9.35%	13.75%	N/A	6.86%
S9 (16-18 mm)[e]	5.02%	7.36%	6.42%	21.06%	23.17%	13.99%	11.82%	N/A	11.17%
S10 (18-20 mm)[e]	10.09%	6.20%	3.20%	28.86%	16.70%	15.92%	4.86%	N/A	14.18%
Pustular mat (0-10 mm)[f]	72.67%	0.02%	0.01%	0.26%	18.56%	6.46%	0.35%	1.21%	0.45%
P1 (0-2 mm)[g]	63.91%	0.06%	0.01%	0.64%	28.95%	4.39%	0.27%	1.22%	0.55%
P2 (2-4 mm)[g]	76.16%	0.01%	0.01%	0.15%	14.69%	8.13%	0.19%	0.38%	0.28%
P3 (4-6 mm)[g]	83.42%	0.04%	0.00%	0.08%	12.02%	3.22%	0.28%	0.66%	0.28%
P4 (6-8 mm)[g]	74.94%	0.01%	0.00%	0.26%	17.22%	4.32%	0.26%	2.62%	0.37%
P5 (8-10 mm)[g]	64.91%	0.00%	0.01%	0.19%	19.92%	12.26%	0.77%	1.15%	0.79%

[a]Relative abundance calculated as the average archaeal composition of the layers, using clustered OTUs at 97% cut-off of archaeal 16S rDNA
[b]Parvarchaeal order YLA114 and WCHD3-30 are the only two orders of class Parvarchaea
[c]Others include archaeal classes Thermoprotei, Methanococci, Thermococci, Methanobacteria, Aigarchaeota, Nanoarchaeoti, DSEG, MCG, MHVG and other Thaumarchaeota (excluding genus *Nitrosopumilus*)
[d]Relative abundance of each class for the whole mat (smooth)
[e]Relative abundance of each discrete 2-mm layer in smooth mats (S1-S10)
[f]Relative abundance of each class for the whole mat (pustular)
[g]Relative abundance of each discrete 2-mm layer in pustular mats (P1-P5)
